# Exploration and Characterization of Novel Glycoside Hydrolases from the Whole Genome of *Lactobacillus ginsenosidimutans* and Enriched Production of Minor Ginsenoside Rg3(*S*) by a Recombinant Enzymatic Process

**DOI:** 10.3390/biom10020288

**Published:** 2020-02-12

**Authors:** Muhammad Zubair Siddiqi, Sathiyaraj Srinivasan, Hye Yoon Park, Wan-Taek Im

**Affiliations:** 1 Department of Biotechnology, Hankyong National University, 327 Jungang-ro Anseong-si, Gyeonggi-do 17579, Korea; mzsiddiqi1988@gmail.com; 2 AceEMzyme Co., Ltd., Academic Industry Cooperation, 327 Jungang-ro Anseong-si, Gyeonggi-do Anseong-si, Gyeonggi-do 17579, Korea; 3 Department of Bio & Environmental Technology, Division of Environmental & Life Science, College of Natural Science, Seoul Women’s University, 623 Hwarangno, Nowon-gu, Seoul 139-774, Korea; sathiya.micro@gmail.com; 4 National Institute of Biological Resources, Incheon 22689, Korea; rejoice077@korea.kr

**Keywords:** *Lactobacillus ginsenosidimutans*, complete genome sequence, novel glycoside hydrolases, bioconversion, recombinant enzyme, ginsenoside Rg3(*S*), gram unit production

## Abstract

Background: Several studies have reported that ginsenoside Rg3(*S*) is effective in treating metastatic diseases, obesity, and various cancers, however, its presence in white ginseng cannot be estimated, and only a limited amount is present in red ginseng. Therefore, the use of recombinant glycosidases from a Generally Recognized As Safe (GRAS) host strain is a promising approach to enhance production of Rg3(*S*), which may improve nutritional activity, human health, and quality of life. Method: *Lactobacillus ginsenosidimutans* EMML 3041^T^, which was isolated from Korean fermented pickle (kimchi), presents ginsenoside-converting abilities. The strain was used to enrich the production of Rg3(*S*) by fermenting protopanaxadiol (PPD)-mix-type major ginsenosides (Rb1, Rb2, Rc, and Rd) in four different types of food-grade media (1, MRS; 2, Basel Food-Grade medium; 3, Basel Food-Grade medium-I, and 4, Basel Food-Grade medium-II). Due to its tendency to produce Rg3(*S*), the presence of glycoside hydrolase in *Lactobacillus ginsenosidimutans* was proposed, the whole genome was sequenced, and the probable glycoside hydrolase gene for ginsenoside conversion was cloned. Results: The *L. ginsenosidimutans* EMML 3041^T^ strain was whole genome sequenced to identify the target genes. After genome sequencing, 12 sets of glycoside hydrolases were identified, of which seven sets (α,β-glucosidase and α,β-galactosidase) were cloned in *Escherichia coli* BL21 (DE3) using the pGEX4T-1 vector system. Among the sets of clones, only one clone (BglL.gin-952) showed ginsenoside-transforming abilities. The recombinant BglL.gin-952 comprised 952 amino acid residues and belonged to glycoside hydrolase family 3. The enzyme exhibited optimal activity at 55 °C and a pH of 7.5 and showed a promising conversion ability of major ginsenoside Rb1→Rd→Rg3(*S*). The recombinant enzyme (GST-BglL.gin-952) was used to mass produce Rg3(*S*) from major ginsenoside Rb1. Scale-up of production using 50 g of Rb1 resulted in 30 g of Rg3(*S*) with 74.3% chromatography purity. Conclusion: Our preliminary data demonstrated that this enzyme would be beneficial in the preparation of pharmacologically active minor ginsenoside Rg3(*S*) in the functional food and pharmaceutical industries.

## 1. Introduction

Natural products (especially secondary metabolites of herbal medicinal plants) play therapeutic roles in treating numerous human diseases. Among these natural products, the biologically active compounds of Panax ginseng are famous around the world because of their strong pharmacological effects against numerous human diseases [1,2,3,4,5,6]. Historically, to produce biologically active minor ginsenosides, the ginseng plant was treated with heat or steam and was commonly known as red or black ginseng. However, in recent years, due to new technology, these two traditional methods (steaming and heating) were replaced by more conventional ginsenoside transformation methods such as bacterial transformation, fungal enzyme transformation, and recombinant enzyme biotransformation [5,7,8]. Due to the presence of several biologically active compounds in ginseng extract, the popularity of ginseng products became not only limited to Asian countries (e.g., South Korea, Japan, China, Vietnam, India, and Pakistan), but recently also gained immense attention in Western countries. More than 80% of ginseng extract is composed of the major ginsenosides Rb1, Rc Rd, Re, and Rg1, which are categorized as protopanaxadiol (PPD)- and protopanaxatriol (PPT)-type ginsenosides based on the structure of the aglycon [9,10]. However, these major ginsenosides can be transformed into pharmacologically active minor ginsenosides, such as Rg3(*S*), F2, C-K, CM-c1, and Rh2, by microorganisms or recombinant enzymes [4,6,7,8]. Minor ginsenosides exhibit various antitumor, antistress, anti-inflammatory, anticancer, immunomodulatory, anti-atherosclerotic, anti-allergic, anti-osteoporosis, antiproliferation, antidiabetic, antigenotoxic, and antihypertensive properties [1,2,3,4,5,6].

Among the minor ginsenosides (e.g., C-Mc1, C-Mx1, Rh2, Rh1, F2, C-K, Rk1, Rg5, Rh3, Rg6, and Rg4), Rg3(*S*) is widely used due to its strong pharmacological effects against several cancers and lung metastasis [11]. Additionally, Rg3(*S*) is known to exert antitumor [12] and anti-obesity effects involving the AMPK signaling pathway and PPAR-c inhibition [13], decrease intracellular calcium levels [14], reduce cytotoxicity in colon cancer, which depends on several mechanisms including apoptosis [15], inhibit prostate cancer cell proliferation [16], and significantly inhibit the growth and angiogenesis of ovarian cancer cells when used alone or in combination with cyclophosphamide [17]. It has been reported that ginsenoside Rg3 prevents the opening of MPTP through free radical-scavenging action in the brain and may contribute to its neuroprotective effect [18]. The protective effects of ginsenoside Rg3 are mainly related to the induction of apoptosis and the inhibition of proliferation, metastasis, and angiogenesis, as presented in Table S1.

Rg3(*S*) is present in very small amounts in total ginseng extract, therefore, two conventional methods (the steaming method and heat acid treatment) were developed to increase production [19]. However, these two practices generate byproducts, such as Rg3(R), Rg2, Rh1, Rk1, Rg5, Rg4, and Rg6, thereby decreasing the concentration of Rg3(*S*). As a result, researchers recently introduced microorganism- and recombinant enzyme-based methods, which are considered to be more suitable processes compared to the steaming method and heat acid treatment for the production of target minor ginsenosides. 

Accordingly, the present study aimed to explore the efficient novel glycoside hydrolases from a Generally Recognized As Safe (GRAS) host, *Lactobacillus ginsenosidimutans* EMML 3041^T^, through whole genome sequencing to enrich and produce ginsenoside Rg3(*S*) up to the gram scale from the PPD-mix-type ginsenosides Rb1 and Rd. 

## 2. Materials and Methods

### 2.1. Materials

The ginsenoside standards, Rb1, Rb2, Rc, Rd, and Rg3(*S*), as well as the PPD mixtures (mainly composed of Rb1, Rb2, Rc, and Rd) were purchased from AceEMzyme Co., Ltd., Republic of Korea. Fifteen different kinds of glucose substrates (with para- (*p*-) and ortho- (*o*-) nitrophenol configurations) as well as the other chemicals used in this study were purchased from Sigma (St. Louis, MO, USA).

### 2.2. Strain Isolation and Fermentation of PPD-Mix-Type Major Ginsenosides 

The strain *L. ginsenosidimutans* EMML 3041^T^, which belongs to the family Lactobacillaceae, was isolated from Korean fermented pickle (kimchi) [20]. The strain was positive for its β-glucosidase activity and was responsible for the hydrolysis of PPD-type major ginsenosides (PPD-mix ginsenosides (Rb1 and Rd)) into minor ginsenosides Rg3(*S*). The PPD-mix-type major ginsenosides, which were extracted from Korean red ginseng, were dissolved in MRS and three different food-grade media (i.e., Basel Food-Grade (BFG), Food-Grade medium I (FG-I) and Food-Grade medium II (FG-II)) [21], with certain modifications, as presented in Table S2. The PPD-mix solutions were sterilized using 0.20 µm ADVANTEC filter paper and *L. ginsenosidimutans* was inoculated in the PPD-mix solutions (PPD-mix in MRS and three food-grade media). Next, the samples were incubated for 1–3 days at 30 °C.

### 2.3. Exploration of Glycoside Hydrolase Gene Through Complete Genome Sequencing

#### 2.3.1. Identification of Target Functional Genes

In the taxonomic characterization of novel bacterial strains and target gene identification, complete genome sequence (CGS) plays a pivotal role in determining a target functional gene at a single time. Therefore, for target functional gene identification, the whole genome of *L. ginsenosidimutans* EMML 3041^T^ was sequenced by Macrogen Korea, and the whole genome sequence was processed as described previously for genome annotation [22]. Moreover, the complete genome sequence was submitted to NCBI GenBank; the project information of *L. ginsenosidimutans* EMML 3041^T^ is available at the Genomes OnLine Database (GOLD). 

#### 2.3.2. NCBI Accession Number 

After CGS analysis, the CGS of *L. ginsenosidimutans* EMML 3041^T^ was deposited into DDBJ/EMBL/GenBank under the accession number CP012034. The strain *L. ginsenosidimutans* was collected from the Korean Agricultural Culture Collection, South Korea, with the accession number KACC 14527^T^ and with the host institution.

### 2.4. Multilocus Sequence Typing for Identification of Species-Specific Genes

Multilocus sequence typing (MLST) is a molecular epidemiology tool which has been used extensively in recent years to differentiate between molecular subtypes of bacteria using whole genome sequencing. MLST is usually based on 6–7 species-specific genes that can be stored in Internet-based databases to easily compare isolates from different geographical areas of the world more conveniently. In the present study, an online web server (Automated Multi-Locus Species Typing) was used to identify species-specific genes of *L. ginsenosidimutans* EMML 3041^T^ to help other researchers compare the genome of the GRAS host *Lactobacillus* species. 

### 2.5. Target Gene Identification, Cloning, and Expression and Biotransformation of Ginsenosides 

The Carbohydrate-Active enZYmes (CAZY) database (http://www.cazy.org/b4765.html) revealed that the glycoside hydrolase genes annotated via NCBI IMG/ER belong to different glycoside hydrolase families (GH1, GH2, GH3, GH13, GH23, GH36, GH65, and GH75). Thus, from the complete genome sequence analysis, a total of 12 glycoside hydrolases (3 *β*-glucosidase, 1 *α*-glucosidase, 2 *α*-galactosidase, 1 *β*-galactosidase, and 1 each of ribose pyranase, galactopyranose mutase, *β*-xylanase, glucosidase, and *β*-D-ribofuranosyl) were identified (Table 1 and Table S3), of which 7 glycoside hydrolases (1 *α*-glucosidase, 3 *β*-glucosidase, 2 β-galactosidase, and 1 *α*-galactosidase) were selected for cloning. A set of forward and reverse (5′–3′) primers (Table 1) were designed by Macrogen Co. Ltd., Korea to target the functional genes. After PCR amplification, the fragments were purified and introduced into the pGEX 4T-1 GST fusion vector using an EzCloning Kit (Enzynomics Co. Ltd., Daejeon, Korea) with BamHI and XhoI restriction sites. After Ezcloning, the resultant recombinants, named AglL.gin-556, BglL.gin-484, BglL.gin-902, BglL.gin-952, AgalL.gin-556, and BgalL.gin-629, were used to transform *Escherischia coli* BL21 (DE3) competent cells and were grown in an LB-ampicillin medium at 37 °C until the culture achieved an OD_600_ value of 0.4–0.6. At this point, the cultures were cooled with an ice water bath and the optimum enzyme activity was attained by induction with various concentrations of 0.1 mM isopropyl-β-d-thiogalactopyranoside (IPTG); the cells were the further incubated for 18 h at 25 °C. After 24 h, the cells were collected via centrifugation and were washed twice with 1% Triton solution. After washing, the pellets were dissolved in 100 mM sodium phosphate (pH 7.0) and were ultrasonicated (Branson Digital Sonifier 450, CT, Danbury, Mexico) to obtain intercellular enzymes. To obtain the crude soluble enzymes, undesirable cell debris was removed by centrifugation (8000 rpm for 15 min at 4 °C). The recombinant enzyme activity and expression were confirmed by hydrolysis of 16 different glucose substrates, as listed in Table S4. SDS-PAGE analysis was performed, which revealed that the molecular weights of the well-localized proteins in the gels were the same as those calculated via amino acid sequence analysis. 

**Figure 1:**
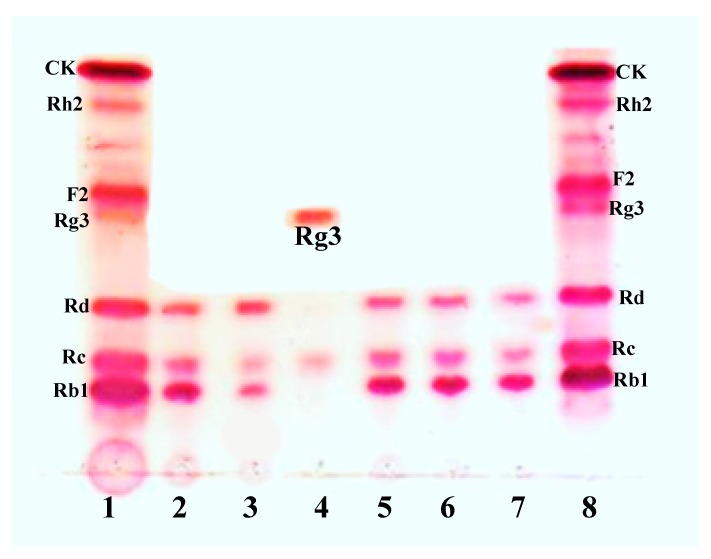
Conversion of protopanaxadiol (PPD)-mix-type ginsenosides by seven different glycoside hydrolases. 1, Ginsenoside standard; 2, AglL.gin-556; 3, BglL.gin-484; 4, BglL.gin-952; 5, BglL.gin-902; 6, AgalL.gin-556; 7, AgalL.gin-319; 8, ginsenoside standard.

The initial biotransformations of seven recombinant enzymes (AglL.gin-556, BglL.gin-484, BglL.gin-902, BglL.gin-952, AgalL.gin-556, AgalL.gin-319, and BgalL.gin-629) were carried out using the PPD-mix-type major ginsenosides Rb1, Rc, and Rd. However, this analysis revealed that only one recombinant enzyme (BglL.gin-952) was responsible for the conversion of the PPD-mix-type of major ginsenosides into minor ginsenosides, as illustrated in Figure 1. Furthermore, the recombinant BglL.gin-952 was characterized and used for the gram unit production of ginsenoside Rg3(*S*) from Rb1. 

#### Phylogenetic Analysis of BglL.gin-952

To identify the exact position of BglL.gin-952 in the different glycoside hydrolase families (e.g., GH1, GH2, and GH3), a database homology search was performed using the BLAST program provided by NCBI, and the sequences of the characterized glycosyl hydrolases were obtained from the CAZY database (http://www.cazy.org). Multiple alignments were performed using the CLUSTAL_X program [23], gaps were edited in the BioEdit program [24], and evolutionary distances were calculated using the Poisson model. A phylogenetic tree was constructed using the neighbor-joining algorithm [25] obtained from the MEGA6 Program [26], with bootstrap values based on 1000 replicates [27].

### 2.6. Characterization of Novel Recombinant Bgll.Gin-952 and Purification

Optimum enzyme activity (recombinant BglL.gin-952) was determined by culturing the recombinant *Escherichia coli* in LBA (Luria-Bertani supplemented with 100 mg/mL ampicillin) broth and incubating the flasks at 37 °C. After the OD_600_ reached 0.4, the optimum induction of recombinant BglL.gin-952 was determined with various concentrations (0.1, 0.5, and 1 mM) of IPTG (Isopropyl β-D-1-thiogalactopyranoside) at 28 °C. After IPTG optimum induction, the effects of various temperatures (18 °C, 22 °C, 25 °C, and 28 °C) were also identified for optimum enzyme activity using the optimum induced concentration of IPTG (0.1 mM). After 18 h of incubation at 18 °C, 22 °C, 25 °C, and 28 °C, the cells were harvested and washed twice with 1% Triton solution and the pellets (10 g/100 mL) were dissolved in 50 mM sodium phosphate buffer (pH 7.0). Enzymes were extracted from the cells using the Branson Digital Sonifier (400 W, 70% power, Branson Digital Sonifier 450, CT, Danbury, Mexico) at the following settings: low power (36%) output, 2 s ON/2 s OFF pulses, and ±4 °C ice-water bath. The total sonication time was 5 min. The crude cell extract was retained and the unwanted cell debris was removed by centrifugation (5000 rpm for 10 min at 4 °C). The GST tag was purified using GST·Bind agarose resin (Elpisbiotech Co. Ltd., Korea), and the homogeneity of the protein was assessed using 8% and 10% SDS-PAGE and an EZStain Aqua solution (ATTO Corporation, Tokyo, Japan). The SDS-PAGE analysis revealed the exact molecular weights of the well-localized proteins as presented by the amino acid sequence analysis (131 kDa).

#### 2.6.1. Effect of pH, Temperature, and Metal Ions on Recombinant Enzyme Activity

To identify the specific enzyme activity of BglL.gin-952, the samples were incubated in 50 mM sodium phosphate buffer (pH 7.0), with *p*-nitrophenyl-*β*-d-glucopyranoside (*p*NPG) as a surrogate substrate for 15 min at 37 °C. After 15 min, the reaction was stopped with 0.5 M (final concentration) Na_2_CO_3_ and the amount of *p*-nitrophenol released was immediately measured by a microplate reader at 405 nm (Bio-Rad model 680; Bio-Rad, Hercules, CA, USA). The amount of recombinant enzyme required to generate 1 μmol of *p*-nitrophenol in one min is known as one unit of enzyme activity. The specific activity of the enzyme was expressed as units per milligram of enzyme. The protein concentration was determined using the Bio-Rad protein assay (catalog number 500-0006) with bovine serum albumin (Sigma, St. Louis, Missouri, USA) as the standard. All assays were performed in triplicate.

In general, enzymes are quite sensitive to environmental conditions and even small changes in the environment may markedly affect enzyme activity. Thus, in this study, we investigated the effect of pH, temperature, and some commonly known metal ions and chemicals.

The effect of pH on the enzyme activity of BglL.gin-952 was confirmed using various types of buffer solutions. Briefly, 1.0 mM *p*NPG was used a substrate in the following buffers with a final concentration of 100 mM: KCl–HCl and glycine–HCl or citrate were used for pH 2.0 and 3.0, respectively, sodium acetate and sodium phosphate were used for pH 4.0–7.5, and Tris–HCl and glycine–sodium hydroxide or carbonate were used for pH 8.0–10.0. Similarly, pH stability was determined as previously stated by Siddiqi et al. [22]. To investigate the effect of optimum temperature on the enzyme activity of BglL.gin-952, the samples were incubated at optimum pH for 10 min in 100 mM sodium phosphate buffer with 2.0 mM *p*NPG under various temperatures (5 °C–80 °C, with intervals of 2 °C (e.g., 35 °C, 37 °C) and 5 °C). As Bgll.gIN-952 was extremely sensitive to temperature, the thermostability of the enzyme was observed by incubating the samples in 100 mM sodium phosphate buffer for different periods of time (5 min, 10 min, 30 min, and 1 h) at various temperatures (5.0–80 °C). After the preset time intervals, the samples were collected and enzymatic activity was determined using *p*NPG as the substrate. 

Several studies have reported that naturally occurring elements, such as metals and heavy metals at various concentrations, affect enzyme activity. Therefore, high concentrations of heavy metals are toxic to enzyme active sites and may inhibit activity. Thus, the effects of metals and other chelating agents or chemicals on the enzyme activity of BglL.gin-952 were determined. BglL.gin-952 activity was evaluated in the presence of 1 and 10 mM (as the final concentration) of KCl, CaCl_2_, CoCl_2_, NaCl, MgCl_2_, MnSO_4_, MgSO_4_, EDTA, and *β*-mercaptoethanol for 15 min at 55 °C. Next, enzyme activity was determined using *p*NPG as a substrate, with the results expressed as a percentage of the activity in the absence of metals and other chemicals.

Typically, all enzyme types prefer to hydrolyze specific substrates and do not yield positive results for the hydrolysis of other substrates. Therefore, in this study, the substrate preference of BglL.gin-952 was determined using 2.0 mM (as final concentration) of 15 different sugar substrates (all from Sigma, as presented in Table S5) with *o*-nitrophenyl (*o*NP) and *p*-nitrophenyl (*p*NP) configurations. Next, the samples were incubated at 37 °C (stable temperature) and 55 °C (optimum temperature) for 5 and 10 min, and enzymatic activity was observed. The amount of enzyme required to release 1 μmol of *o*NP or *p*NP in 1 min is known as one unit (U) of enzyme.

#### 2.6.2. Enzymatic Transformation of Major Ginsenosides by Bgll.Gin-952

To assess the effect of the fused GST tag on the recombinant enzyme activity of BglL.gin-952, the initial biotransformation of Rb_1_, Rd, and Re was tested. The analysis revealed that the fused GST tag did not affect the activity of the recombinant BglL.gin-952, thus, the GST-fused enzyme (GST-BglL.gin-952) was used to determine the specificity and selectivity of BglL.gin-952 for removing glucose molecules attached to the C3, C6, and C20 positions in four PPD-type (Rb_1_, Rb_2_, Rc, and Rd) and two PPT-type (Re and Rg_1_) major ginsenosides. An equal volume of enzyme solution (concentration of 0.5 mg mL^−1^) in 100 mM of sodium phosphate buffer (pH 7.0) was mixed with Rb_1_, Rb_2_, Rc, Rd, Re, and Rg_1_ at a concentration of 1000 ppm at 37 °C for 24 h. After regular time intervals, the samples were collected and analyzed by thin-layer chromatography (TLC) to evaluate the enzymatic conversion of the PPD- and PPT-type ginsenosides.

### 2.7. Chromatography

#### 2.7.1. TLC analysis

Thin-layer chromatography (TLC) was performed using 60F254 silica gel plates (Merck, Darmstadt, Germany) with CHCl_3_–CH_3_OH–H_2_O (65:35:10, lower phase) as the solvent. The spots on the TLC plates were visualized by spraying H_2_SO_4_ (10%, v/v) and heating at 110 °C for 5 min. The results were compared with the ginsenoside standards.

#### 2.7.2. HPLC analysis

HPLC analysis of the ginsenosides was carried out using an HPLC system (Young Lin Co. Ltd., Anyang, Korea). Ginsenoside separation and peaks were achieved using a C_18_ column (5 μm, 150 × 4.6 mm i.d.; Phenomenex, Torrance, California, United States) with a guard column (Eclipse XDB C_18_, 5 μm, 12.5 × 4.6 mm i.d.). The mobile phases were water (A) and acetonitrile (B) with a gradient as follows: 0–11 min, 32%–65% B; 12–18 min, 100% B; and 15–26 min, 32% B. The flow rate was maintained at 1.0 mL/min and the sample injection volume was 25 µL. Detection was performed by monitoring the absorbance at 203 nm. 

## 3. Results

### 3.1. Bioconversion of PPD Mixtures by L. Ginsenosidimutans EMML 3041^T^

**Figure 2:**
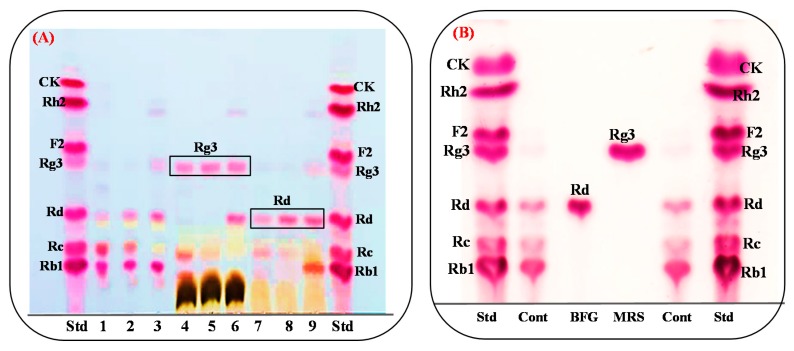
(**A**) Thin-layer chromatography (TLC) analysis showed the conversion of the PPD-mix extracts by *Lactobacillus ginsenosidimutans* EMML 3041^T^ from Korean ginseng, American leaf saponins, and Chinese ginseng. Std, ginsenosides standards; 1. Korean ginseng extract (KGE) control; 2, American leaf extract (ALE) control; 3, Chinese ginseng extract (CGE) control; 4–6, conversion of KGE, ALE, and CGE in MRS broth; 7–9, conversion of KGE, ALE, and CGE in Basel Food-Grade broth. (**B**) The effect of two different media (MRS and BFG) on the bioconversion of PPD-mix-type ginsenosides. Std, ginsenoside standard; cont, control of PPD-mix ginsenosides (Rb1, Rc, and Rd).

The strain *L. ginsenosidimutans*, which was isolated from Korean fermented pickle (kimchi), efficiently converted the major ginsenosides Rb1 and Rd to the minor ginsenoside Rg3(*S*) in MRS medium. For the edible and economic production of Rg3(*S*), three different types of food-grade media (BFG, FG-1, and FG-II) were used. Briefly, *L. ginsenosidimutans* was grown in BFG, FG-1, and FG-II broths and the growth of culture of the food-grade media were compared with the strain grown in MRS medium (broth). The results indicated that BFG medium was more suitable for the growth of *L. ginsenosidimutans* compared to FG-I and FG-II media (as presented in Figure S1), therefore, this medium was selected for the bioconversion of major ginsenosides into minor ginsenosides. Interestingly, the strain did not produce Rg3(*S*) in the BFG medium, but the initial bioconversion of the major ginsenosides (which were extracted from Korean red ginseng, American leaf saponins, and Chinese ginseng) revealed that the *L. ginsenosidimutans* powerfully transformed the PPD-mix ginsenosides to Rd and the target minor ginsenoside Rg3(*S*) in the BFG and MRS media, respectively (Figure 2A,B). Thus, based on the initial fermentations and quality of the ginseng extracts (Korean, American, and Chinese ginseng extracts), the American leaf saponins were selected for the edible production of the target minor ginsenoside Rg3(*S*) (Figure 2B). The ginsenoside bioconversion pathways for *L. ginsenosidimutans* in two different media are presented in Figure S2.

### 3.2. Genome Properties

The complete genome of *L. ginsenosidimutan* EMML 3041^T^ was a presumptive circular chromosome of 2,590,556 base pairs (Figure S3) with a G + C content of 36.71%. A total of 2574 genes, 2486 CDS, and 62 RNA genes were assigned a putative function, and the remainder were annotated as hypothetical or conserved hypothetical proteins (Table 2). The total counts of different functional genes and predicted COG and KOG functional genes are depicted in Figure S3 and Table S6. Based on the complete genome annotation, it was evident that the genome comprised numerous glycoside hydrolase metabolism genes clusters, including β-glucosidase, which may be responsible for ginsenoside conversion. Furthermore, from the whole genome analysis, a total of 58 (10.38%) genes were annotated as being involved in carbohydrate transport and metabolism (Table S6).

The auto-MLST revealed 87 characterized, known and uncharacterized, or unknown species-specific genes, which are listed in Table S7. The main advantage of this technique was that it provided unambiguous data that are reproducible among laboratories. For individual species-specific (or housekeeping) genes, the different sequences present within a bacterial species were assigned as distinct alleles; for each isolate, the alleles at each of the loci defined the allelic profile or sequence type (ST).

### 3.3. Phylogenetic Analysis of Sequence of BglL.Gin-952

**Figure 3:**
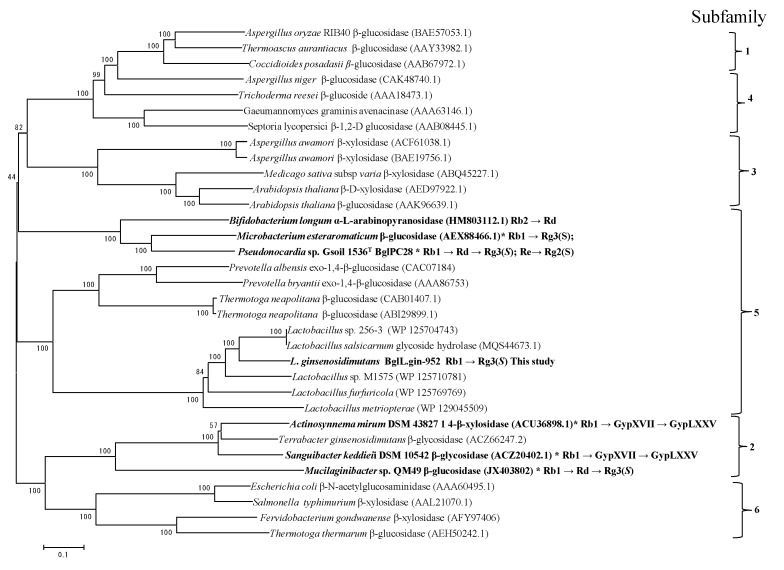
Phylogenetic tree analysis showing the position of the novel BglL.gin-952 in the glycoside hydrolases of family 3 (GH3). The tree was constructed using the maximum-likelihood algorithm alongside a Poisson model and pairwise deletion. Bootstrap values expressed as percentages of 1000 replications greater than 50% are shown at the branch points. The bar represents 10 amino acid residue substitutions per 100 amino acid residues.

The β-glycosidase gene (BglL.gin-952, GenBank accession number: WP_053084464) comprised 2859 bp encoding 952 amino acids with a molecular mass of 131.0 kDa. The hypothetical pI value of of BglL.gin-952 is 4.89 (http://web.expasy.org/compute_pi/). Amino acid sequence analysis of BglL.gin-952 indicated that it possesses less than 82.4% similarity to the glycoside hydrolase of *Lactobacillus* sp. 256–3 (GenBank number WP_125704743) and other uncharacterized glycoside hydrolases, suggesting that BglL.gin-952 has a domain similar to glycoside hydrolase family 3 (GH3). However, GH3 is subdivided into several subfamilies. In order to identify the evolutionary position of BglL.gin-952 within the characterized enzymes in GH3, a phylogenetic analysis was constructed using the maximum-likelihood method from the MEGA-6 Program with bootstrap values of 1000 replications. This analysis revealed that BglL.gin-952 was clustered within subfamily 5 in GH3, forming a separate but well-supported group with the β-glucosidase derived from other *Lactobacillus* species (Figure 3). 

### 3.4. Molecular Cloning

The oligonucleotide primer sequences used for gene cloning were based on the DNA sequences of the aforementioned genes, as listed in Table 2. The primers were designed and synthesized by Macrogen Co. Ltd. (Seoul, Korea) with BamHI and XhoI restriction sites.

After functional gene cloning, a few sets of the clones presented positive and weakly positive activities for *p*NP-β-d-glucopyranoside, pNP-β-d-galactopyranoside, oNP-β-d-glucopyranoside, and oNP-β-d-galactopyranoside. Among these seven sets of clones, only one gene (BglL.gin-952) was responsible for the conversion of the ginsenosides (Table S4). 

### 3.5. Protein Expression and Biotransformation of Ginsenosides by Bgll.Gin-952

**Figure 4:**
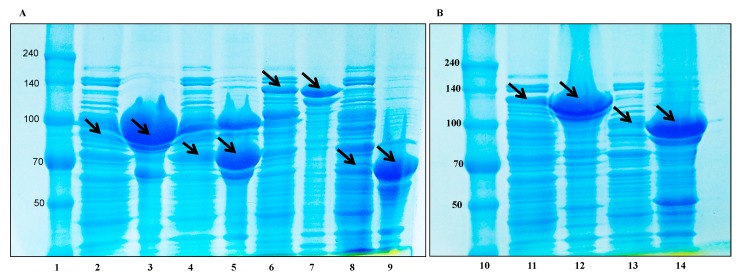
(**A**,**B**). SDS-PAGE analysis of recombinant glycoside hydrolase genes of *L. ginsenosidimutans*. Lanes 1 and 10, protein marker (50–240 kDa); Lanes 2 and 3, soluble and precipitate enzyme of AglL.gin-556 (87 kDa), respectively; Lanes 4 and 5, soluble and precipitated enzymes of BglL.gin-484 (79 kDa), respectively; Lanes 6 and 7, soluble and precipitated enzymes of BglL.gin-952; Lanes 8 and 9, soluble and precipitated enzymes of AgalL.gin-319, respectively.

**Figure 5:**
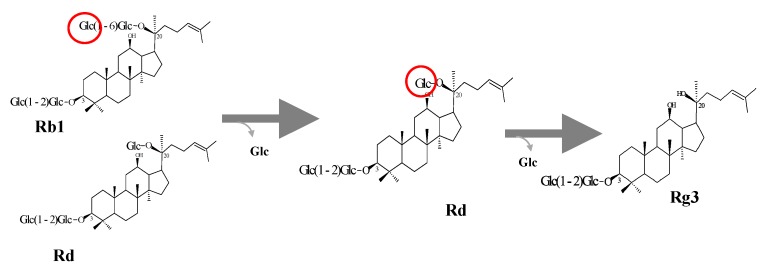
Conversion pathway of ginsenosides Rb1 and Rd to Rg3(*S*) by recombinant BglL.gin-952.

The recombinant pGEX- AglL.gin-556, BglL.gin-484, BglL.gin-952, BglL.gin-902, AgalL.gin-556, and AgalL.gin-319 enzymes were used to transform *E. coli* BL21 (DE3). To maximize the yield and expression of the fusion protein, the protein was induced by adding 0.1 mM IPTG. The clone samples of the recombinant *E. coli* BL21 (DE3) grown in LBAmp (1 g mL^−1^) broth were preserved at different temperatures (18 °C, 22 °C, 25 °C, and 28 °C) with varying induction conditions (0.05, 0.1, and 0.5 mM IPTG (data not shown)). Incubation with 0.1 mM IPTG at 18 °C for 18 h produced the maximum level of soluble, active, fused enzyme, as depicted in Figure 4A,B. The ability of recombinant pGEX- AglL.gin-556, BglL.gin-484, BglL.gin-952, BglL.gin-902, AgalL.gin-556, and AgalL.gin-319 to convert PPD-type ginsenosides (Rb1, Rb2, Rc, and Rd) was assessed by performing TLC analysis of the enzyme hydrolysates at regular intervals. Based on the Rf values, it was evident that BglL.gin-952 converted the PPD-type (Rb1 and Rb2) major ginsenosides into Rg3(*S*) (Figure 1). Thus, the results indicated that only the BglL.gin-952 efficiently hydrolyzed the glucose attached to the C3 and C20 positions of the PPD-type ginsenosides. The proposed pathway for the biotransformation of the PPD-type ginsenosides (2 mg mL^−1^) by BglL.gin-952 is Rb1 and Rd to Rg3(*S*), where the conversion of PPD-mix type ginsenosides occurs through the stepwise hydrolysis of the inner and outer glucose attached at the C3 and C20 positions of aglycon (Figure 5). Furthermore, the incubation of Rg3(*S*) along with the recombinant enzyme (BglL.gin-952) did not lead to hydrolysis of Rg3(*S*), indicating that the recombinant BglL.gin-952 preferred the outer and inner glucose moiety at the C20 position.

#### Characterization of BglL.Gin952

The cell lysate of the recombinant BglL.gin-952 was purified via the GST·bind agarose resin, and the purified protein was subjected to 8% and 10% SDS-PAGE analysis, which confirmed that the exact molecular weight was presented by the full-length sequence (131 kDa), as depicted in Figure S4. The molecular weight (131.0 kDa) of the recombinant protein BglL.gin-952, which was calculated from amino acid-sequence analysis, was similar to that calculated after protein purification.

**Figure 6:**
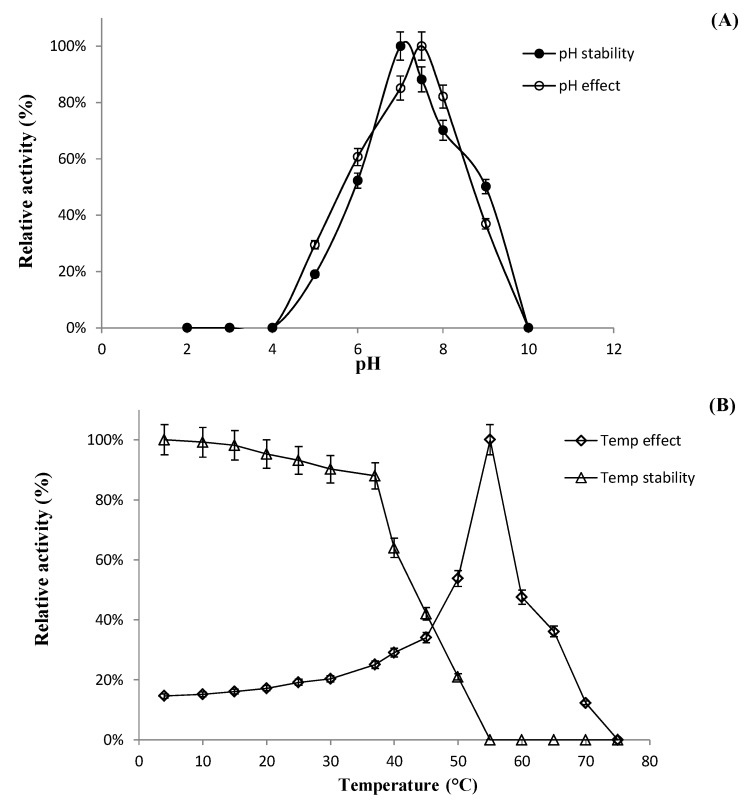
(**A**). Effect of pH on enzyme activity. Enzyme activity was measured under standard assay conditions. Enzyme solutions containing 2.0 mM *p*NPGlc were incubated with various buffers at pH 2.0–10.0 for 12 h at 4 °C. To assess the stability of BglAg-762, the enzymes were incubated for 30 min at 50 °C in various buffers at pH 2.0–10.0, and the residual activities were measured. (**B**) The effect of temperature on the stability and activity of recombinant BglAg-762 was measured under standard assay conditions. The thermodependence of BglAg-762 was assayed using 50 mM potassium phosphate buffer (pH 7.5) at varying temperatures ranging from 4–60 °C. Thermostability was tested by incubating aliquots of the enzyme in 50 mM potassium phosphate buffer (pH 7.5) for different periods of time at various temperatures. After cooling the sample, the residual activity was determined.

The optimum temperature and pH values for the bioconversion of the PPD-mix-type major ginsenosides were determined using a crude recombinant BglL.gin-952 enzyme, which exhibited optimal activity at pH 7.5 in sodium phosphate buffer and stability at pH 6.0–8.0 (Figure 6A). When the pH was less than 6.0 or more than 8.0, the enzyme stability decreased markedly, and at pH 4.0 and 10.0, the enzyme activity decreased rapidly to 0% of the optimum activity (Figure 6A). The optimal temperature was found to be 55 °C, whereas the thermostability decreased significantly below 37 °C and the enzyme activity reached 0% when it was further incubated at 55–75 °C for 2 h (Figure 6B). BglL.gin-952 was stable below 37 °C, and about 60% of the activity was lost after incubation at 45 °C for 1 h. Thus, the optimal temperature for the activity of BglL.gin-952 was higher than that of *β*-glucosidase (45 °C), which was isolated from *Arachidicoccus ginsenosidimutans* and *Terrabacter ginsenosidimutans*, but similar to that of *β*-glucosidase from *Pyrococcus furiosus* (55 °C), indicating that BglL.gin-952 is mesophilic and stable at a neutral pH range. In addition, the near-neutral optimal pH and the mild optimal temperature of BglL.gin-952 were similar to those of ginsenoside-hydrolyzing GH3 from other bacteria [28,29,30]. Even though the optimum temperature of BglL.gin-952 against *p*NPGlc was 55 °C, during prolonged and stable transformation, the ginsenoside bioconversion reaction occurred at 37 °C, with an optimum pH of 7.0–7.5.

As mentioned previously, heavy metals are atoms with high molecular weights, and changes in metal concentrations may affect enzyme activity. Therefore, the effects of various metal ions and some chemicals (EDTA and *β*-mercaptoethanol) on the activity of BglL.gin-952 were investigated. The results were expressed as percentages of activity in the absence of the test compound (Table 3). The activity of BglL.gin-952 was not affected by 1 and 10 mM of metal ions (Na^+^, K^+^, Mg^2+,^ Mn^+^, Co^2+^, Mn^2+^, and Mg^2+^) or chemicals (EDTA and *β*-mercaptoethanol); however, enzyme activity was enhanced by the addition of 10 mM Ca^2+^, Co^2+^, and Mn^2+^ ions. Thus, the BglL.gin-952 recombinant enzyme was found to be less affected by metal ions and chelating agents compared to our previously described recombinant enzyme [30].

The substrate specificity of BglL.gin-952 was investigated with *α*- and *β*-configurations of 1.0 mM *p*NP- and *o*NP-glycosides. The analysis revealed that BglL.gin-952 exhibited the highest activity against *p*NP-*β*-D-glucopyranoside (100.0%), weak activity against *p*NP-β-D-galactopyranoside and pNP-α-L-arabinopyranoside, and no effect on any of the other *p*NP- and *o*NP-glycosides (Table 4).

### 3.6. Scaled-Up Biotransformation of Rg3(S)

#### 3.6.1. Preparation of High-Cell Density Culture of Recombinant Bgll.Gin-952 

A high-cell density culture of the recombinant *E. coli* BL21 (DE3) harboring the pGEX-4T-BglL.gin-952 gene was obtained by inoculating the strain in 5 L of LBA broth (LB broth containing 100 mg ampicillin/mL as the final concentration) at 37 °C and 500 rpm in a 10 L stirred-tank reactor (Biotron GX, Hanil Science Co., Incheon, Korea). The pH of the medium was adjusted to 7.0 using 0.1 M sodium phosphate buffer. When the OD_600nm_ reached 3.0, the culture was allowed to cool, and the protein was induced by 0.1 mM IPTG (final concentration). The culture was further incubated at 18 °C along with glucose (with a final concentration of 10 g/L) until the cell density reached to an OD of 20~25 at 600 nm. The cells were then harvested via centrifugation at 8000 rpm for 10 min and the enzyme was extracted using a Branson Digital Sonifier (Danbury, Mexico). Unwanted cell debris was removed by centrifugation at 4 °C, and the crude soluble enzyme fraction was used for the conversion of ginsenoside Rb1 to Rg3(*S*).

#### 3.6.2. Gram Unit Production of Rg3(*S*)

**Figure 7:**
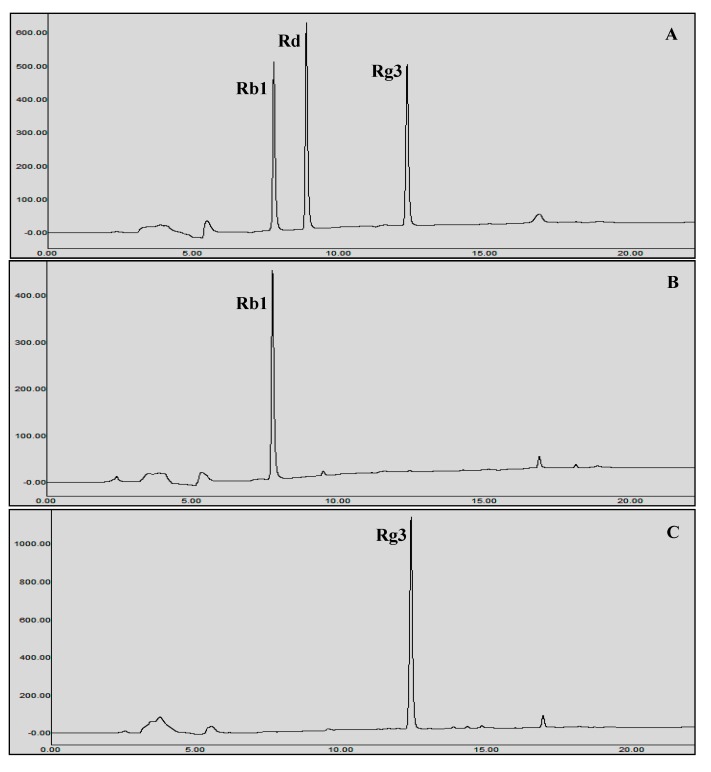
HPLC analysis of the transformation of Rb1 to Rg3(*S*) by recombinant BglL.gin-952. **A**, Ginsenoside standard; **B**, Rb1 as a starting substrate; **C**, Rg3(*S*) production after 24 h of reaction.

**Figure 8:**
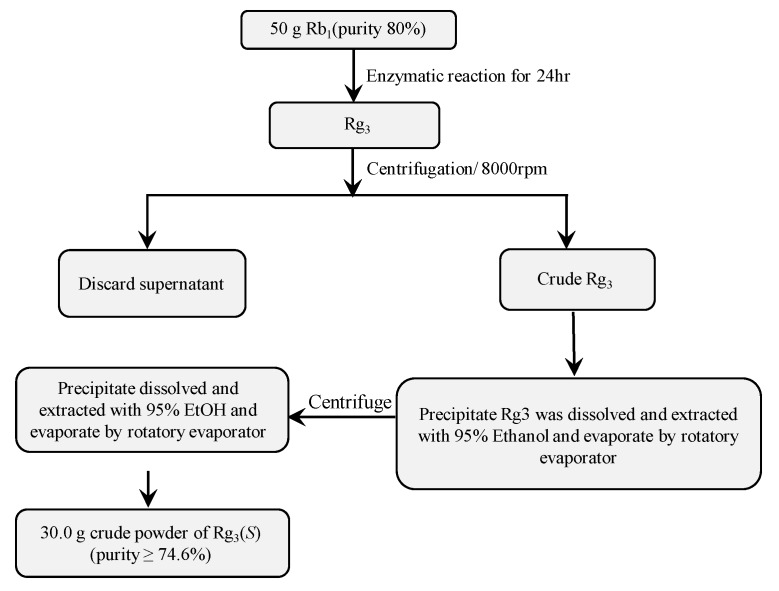
Gram unit production of ginsenoside Rg3(*S*) from ginsenoside Rb1.

To enhance the production of ginsenoside Rg3(*S*), the reaction (Rb1 + BglL.gin-952) was performed in a 10 L stirred-tank reactor with 5 L of working volume. The reaction mixture was prepared with equal volumes (1:1 v/v) of recombinant enzyme solution (30 mg/mL of crude recombinant BglL.gin-952 in 0.1 M of sodium phosphate buffer (pH 7.0 ± 0.2)) and 10 mg/mL (final concentration) of ginsenosides Rb1 (total 50 g). The reaction was completed under optimal conditions at 37 °C and pH 7.0 ± 0.5 at 350 rpm for 24 h. Samples were collected at regular intervals and analyzed using TLC and HPLC to determine the time-course of the biotransformation of ginsenoside Rb1 to Rg3(*S*). After 24 h, Rb1 was completely converted to ginsenoside Rg3(*S*) (Figure 7). The unwanted substances from the reaction mixture were removed by centrifugation at 8000 rpm for 10 min, and Rg3(*S*) was precipitated to a solid form. The precipitated Rg3(*S*) was then dissolved twice in 3 L of a 95% ethanol solution and the Rg3(*S*) supernatant was evaporated in a vacuum to obtain 30 g of powdered Rg3(*S*) (Figure 8, Figure S5).

### 3.7. Comparision of Rg3(S) Production between Bacterial Cell Fermentation and the Recombinat Enzyme Bgll.Gin952

In the comparative analysis of ginsenosude Rg3(*S*) production, the PPD-mix (Rb1, Rc, and Rd) was dissolved in MRS medium for cell fermentation and in sodium phosphate buffer (pH 7.0) for the recombinant BglL.gin-952. Ginsenoside fermentation and the enzymatic reactions commenced at a final concentration of 5 mg/mL (total, 5 g/L). The samples were incubated at 30 °C for whole-cell fermentation and 37 °C for the enzymatic reactions. The results indicated that the production of Rg3(*S*) using whole bacterial strain cells was 1.1 g with 53.1% purity after 72 h of incubation and 3.0 g after 24 h for the enzymatic reactions, as shown in Table S8.

## 4. Discussion 

In oriental herbal medicinal plants, red ginseng is a very popular health-promoting food but contains only approximately 1% of the minor ginsenoside Rg3(*S*) based on its dry weight. Although Rg3(*S*) has strong anticancer, antimetastatic, and anti-obesity effects, the lack of selective mass-production technology has slowed down its commercial use. To scale-up the production of Rg3(*S*), several researchers have aimed to accomplish biotransformation of major ginsenosides to minor ginsenosides using microorganisms and recombinant enzymes in laboratory settings; however, these researchers only characterized the basic functions of the microorganism and recombinant enzymes separately from the mass production aspects [31,32,33]. As a result, all of the previous studies focused on the conversion of major ginsenosides into minor ginsenosides using derived glycoside hydrolases from host organisms that are not recognized as safe, with most attempts unsuccessful in obtaining the gram unit production of Rg3(*S*). Recently, Li et al. [31] succeeded in converting the major ginsenoside Rb1 to the minor ginsenosides Rg3 and C-K using an expression system of GRAS host organisms (*Lactococcus lactis*), but their findings were still limited to a laboratory-level production of Rg3(*S*).

Upon selecting appropriate experimental organisms for *β*-glucosidase gene, we found a food-grade bacteria for the edible production of Rg3(*S*) through fermentation of PPD-mix (Rb1 an Rd)-type major ginsenosides. Rg3(*S*) is well-known for its strong pharmacological effects, but only a few studies focused on the edible production of Rg3(*S*) using the GRAS host organism or recombinant enzymes [31], and none of the previous studies managed to produce the gram unit of Rg3(*S*) using the derived glycoside hydrolases from GRAS host organisms. Some researchers tried to use heat acid treatment for the gram unit production of Rg3(*S*), but did not succeed due to certain side reactions and the byproduction of various minor ginsenosides, such as Rg3(*R*), Rg5, and Rk1 [30]. 

Thus, our preliminary data demonstrated that *L. ginsenosidimutans* was suitable for the enriched, gram unit production of edible ginsenoside Rg3(*S*) from PPD-mix-type ginsenosides (Rb1 and Rd). The DNA sequence of BglL.gin-952 was obtained from the complete genome sequenced *L. ginsenosidimutans* EMML 3041^T^., showing that BglL.gin-952 is homologous to glycoside hydrolase family 3 based on its amino acid sequence similarities to the glycoside hydrolases (or β-glucosidases) from *Lactobacillus salsicarnum* (82.5%, GenBank accession no. MQS44673.1), *Lactobacillus* sp. 256–3 (82.4%, GenBank accession no. WP 125704743.1), and *Lactobacillus*. sp M1575 (73.2%, WP 125710781.1). The β-glucosidases have been mainly characterized in glycosyl hydrolase families 1, 3, and 51 (GH1, GH3, and GH51), which usually form in closely related subfamilies with a varied range of activities. Previous research showed that some β-glucosidases of these families may have good hydrolysis specificity to only glucosides or glucosides attached to other monomers, such as β-galactosides, fucosides, β-xylosides, and β-mannosides [34]. Furthermore, some researchers demonstrated that the β-glucosidases belonging to GH3 have various types of ginsenoside-hydrolyzing pathways compared to glycoside hydrolase family 1 and family 51 (GH1 and GH51). The characterized β-glucosides in family 1 and family 51 enable the transformation of major ginsenoside Rb1 → Rd, while the β-glucosides belonging to glycoside hydrolase family 3 convert major ginsenoside Rb1 → Gyp-XVII → Gyp-LXXV [35] and Rb1→ Rd → Rg3(*S*) [33]. Thus, the glycoside hydrolases belonging to family 3 have better potential to make various kinds of minor ginsenosides from major ginsenosides.

As a glycoside hydrolase family 3 enzyme, BglL.gin-952 showed promising ginsenoside-transforming abilities. Furthermore, 12 novel glycoside hydrolases were identified through complete genome sequencing of *L. ginsenosidimutans* EMML 3041^T^ (Table 1 and Table S3), which is a presumptive circular chromosome of 2,590,556 base pairs with a G + C content of 36.71%. The online web server of multilocus sequence typing showed 87 characterized and uncharacterized species-specific genes. Based on complete genome annotation, the 10.38% of this genome is made up of carbohydrate transport and metabolism gene clusters, including β-glucoside and cellobiose operons. Moreover, among 12 glycoside hydrolases (as discussed in Section 2.5), seven genes were cloned and expressed in *E. coli* BL21 (DE3). All seven clones were positive for the substrate metabolism (*p*NPG), however, only one gene (BglL.gin-952) was positive ginsenoside conversion (Table S4). BglL.gin-952 belongs to subfamily 5 of GH3 and was responsible for the gram unit production of Rg3(*S*) from the major ginsenosides Rb1 and Rd. The optimum reaction condition for BglLgin-952 was 55 °C at a pH of 7.5. During conversion, BglL.gin-952 efficiently hydrolyzed the glucose attached to the C3 and C6 positions. 

## 5. Conclusions

To the best of our knowledge, this is the first study in which 30 g of Rg3(*S*) (with 74.3% chromatography purity) was produced from 50 g of Rb1 in one step using a novel recombinant glucoside hydrolase, namely, BglLgin-952. Thus, BglL.gin-952 could play a pivotal role in the large-scale production of ginsensoide Rg3(*S*) at an industrial level. Furthermore, our pilot data showed that the enriched production of Rg3(*S*) using a GRAS host and a recombinant enzyme could enhance the health benefits of *Panax ginseng* in either fermented foods or bioconversion processes and pharmaceutical industries. Additionally, the brief description of the complete genome analysis of *L. ginsenosidimutans* provided in this study may be of use in future studies.

## Figures and Tables

**Table 1:** Set of primers (forward (F) and reverse (R)) used to clone the target functional gene.

Sets of Total Clones	Names of Clone Gene	Total Amino Acids	Common Name	Ginsenoside Conversion Activity	Primers Sequences (F, R)	Vector
1	*α*-glucosidase-556	556	(AglL.gin-556)	-	GGTTCCGCGTGGATCCACAAAAAATTGGTGGCAAGGTT_F AACCTTGCCACCAATTTTTTGTGGATCCACGCGGAACC _R	pGEX 4T-1
2	*β*-glucosidase-484	484	(BglL.gin-484)	-	GGTTCCGCGTGGATCCCGCAAAAATGAGTATCCTTATT_F AATAAGGATACTCATTTTTGCGGGATCCACGCGGAACC_R	
3	*β*-glucosidase-902	902	(BglL.gin-902)	-	GGTTCCGCGTGGATCCGAAAGCGAAATGTCACAACGAG_F CTCGTTGTGACATTTCGCTTTCGGATCCACGCGGAACC_R	
4	*β*-glucosidase-952	952	(BglL.gin-952)	+	GGTTCCGCGTGGATCCGATAAAAAACAGAAGAGGATTG_F CAATCCTCTTCTGTTTTTTATCGGATCCACGCGGAACC_R	
5	*α*-galactosidase-556	556	(AgalL.gin-556)	-	GGTTCCGCGTGGATCCTCTGTTAATGTTAATCAAAGTA_F TACTTTGATTAACATTAACAGAGGATCCACGCGGAACC_R	
6	*α*-galactosidase-319	319	(AgalL.gin-319)	-	GGTTCCGCGTGGATCCGATTACACAAATAATAAGTTGC_F GCAACTTATTATTTGTGTAATCGGATCCACGCGGAACC_R	
7	*β*-galactosidase-629	629	(BgalL.gin-629)	-	GGTTCCGCGTGGATCCCAAGCTGATATTAACTGGCTCG_F CGAGCCAGTTAATATCAGCTTGGGATCCACGCGGAACC_R	

**Table 2:** General features of *Lactobacillus ginsenosidimutan* EMML 3041^T^.

Features	Chromosome
Length (bp)	2,590,556
DNA coding region (bp)	2,271,180
G + C content (%)	36.71
Genes	2574
Pseudogenes	26
CDS	2486
rRNA gene	9
tRNA genes	53

**Table 3:** Substrate hydrolysis activity of BglL.gin-952.

Substrates	Relative Activity ± SD (%)
*p*NP-*β*-d-glucopyranoside	100.0 ± 3.3
*p*NP-*β*-d-galactopyranoside	17.0 ± 1.0
*p*NP-*β*-d-fucopyranoside	5.3 ±0.3
*p*NP-*β*-n-glucosaminide	ND
*p*NP-*β*-l-arabinopyranoside	ND
*p*NP-*β*-d-mannopyranoside	ND
*p*NP-*β*-d-xylopyranoside	ND
*p*NP-*α*-d-glucopyranoside	ND
*p*NP-*α*-d-arabinofuranoside	ND
*p*NP-*α*-d-arabinopyranoside	14.5 ± 0.67
*p*NP-*α*-d-mannopyranoside	ND
*p*NP-*α*-d-mannopyranoside	ND
*o*NP-*β*-d-glucopyranoside	ND
*o*NP-*β*-d-fucopyranoside	ND
*o*NP-*α*-d-galactopyranoside	ND

**Table 4:** Effects of 1 and 10 mM β-mercaptoethanol, EDTA, and some metal ions.

Metal Ions or Reagents	Relative Activity ± SD (%) at:	
	1 mM	10 mM
NaCl	80.0 ± 6.7	85.6 ± 1.3
KCl	87.0 ± 0.7	85.3 ± 3.7
CaCl_2_	81.3 ± 6.1	122.1 ± 0.9
MgCl_2_	82.9 ± 0.2	71.6 ± 3.3
CoCl_2_	84.2 ± 0.4	132.0 ± 3.9
MnSO_4_	88.3 ± 4.0	155.5 ± 5.8
MgSO_4_	91.6 ± 2.4	79.8 ± 0.3
*β*-Mercaptoethanol	88.6 ± 3.0	93.9 ± 0.3
EDTA	84.4 ± 3.8	78.7± 3.7
Control	100.0 ± 2.1	100.0 ± 5.1

## Supplementary Materials

The following are available online at https://www.mdpi.com/2218-273X/10/2/288/s1, **Figure S1** Growth profile of L. ginsenosidimutans in MRS and three food grade media [Based Food Grade (BFG), Food Grade-I (FG-I) and Food Grade-II (FG-II)]. Growth curves were performed in triplicate and the average of those measurements is displayed in the graph above; **Figure S2**. Show the conversion of PPD-mix type major ginsenoside (Rb1and Rd) to Rg3(*S*) by *L*ac. *ginsenosidimutans* EMML 3041^T^; **Figure S3** Graphical map of the chromosome of Lactobacillus ginsenosidimutans EMML 3041^T^. From outside to the center: Genes on the forward strand (colored by COG categories), genes on the reverse strand (colored by COG categories), RNA genes (tRNAs, green; rRNAs, red; other RNAs, black), and GC content and GC skew. And the orthologous proteins of Lactobacillus ginsenosidimutans EMML 3041^T^ and distribution of proteins in different KEG/COG categories are plotted. Y-axis represents the number of genes/proteins and X-axis represents the KEG/COG categories; **Figure S4.** SDS page analyses of purified BglL.gin-952. Lane 1, Protein marker; Len 2, Induced BglL.gin-952; Lane 3, purified BglL.gin-952; **Figure S5.** TLC analysis shows the time course conversion of ginsenoside Rb1 to Rg3(*S*) by BglL.gin-952. S, ginsenosides standards; **Table S1.** Anticancer activities of ginsenoside Rg3; **Table S2.** Show the composition of MRS (Difco), Basel Food Grade medium (BFG), Food Grade medium I and Food Grade medium II. All ingredients were used as g/L; **Table S3.** Identification of glycoside hydrolases from the whole genome sequence of L. ginsenosidimutans EMML 3041^T^; **Table S4.** Show the hydrolytic activities of seven glycoside hydrolase clones of L. ginsenosidimutans EMML 3041^T^ for various sugar substrates. 1, α-glucosidase-556; 2, β-glucosidase-484; 3, β-glucosidase-902; 4, β-glucosidase-952; 5, α-galactosidase-556; 6, α-galactosidase-319; 7, β-galactosidase-629. ++, strong positive (98.0 ± 1.2 %); +, positive (87.0 ± 3.1 %); w, weak positive (63.0 ± 2.2 %); –, negative; **Table S5.** Show the different sugar substrate used for the BglL.gin-952; **Table S6.** Total gene count and their function; **Table S7.** The total housekeeping gene identified in the whole genome sequenced L. ginsenosidimutans EMML 3041^T^; **Table S8.** Show the production of Rg3(*S*) using the *Lac. ginsenosidimutans* EMML 3041^T^ and recombinant BglLgin-952.

## References

[1] Jia L., Zhao Y. (2009). Current evaluation of the millennium phytomedicine-ginseng (I): Etymology, pharmacognosy, phytochemistry, market and regulations. Curr. Med. Chem..

[2] Li W., Zhang M., Gu J., Meng Z.-J., Zhao L.-C., Zheng Y.-N., Chen L., Yang G.-L. (2012). Hypoglycemic effect of protopanaxadiol-type ginsenosides and compound K on Type 2 Diabetes mice induced by High-Fat Diet combining with Streptozotocin via suppression of hepatic gluconeogenesis. Fitoterapia.

[3] Siddiqi M.H., Siddiqi M.Z., Ahn S., Kim Y.-J., Yang D.C. (2014). Ginsenoside Rh1 induces mouse osteoblast growth and differentiation through the bone morphogenetic protein 2/runt-related gene 2 signalling pathway. J. Pharm. Pharmacol..

[4] Siddiqi M.Z., Siddiqi M.H., Kim Y.-J., Jin Y., Huq A., Yang D.-C. (2015). Effect of Fermented Red Ginseng Extract Enriched in Ginsenoside Rg3 on the Differentiation and Mineralization of Preosteoblastic MC3T3-E1 Cells. J. Med. Food.

[5] Wong A.S.T., Che C.-M., Leung K.-W. (2015). Recent advances in ginseng as cancer therapeutics: A functional and mechanistic overview. Nat. Prod. Rep..

[6] Baatar D., Siddiqi M.Z., Im W.T., Khaliq N.U., Hwang S.G. (2018). Anti-Inflammatory Effect of Ginsenoside Rh2-Mix on Lipopolysaccharide-Stimulated RAW 264.7 Murine Macrophage Cells. J. Med. Food.

[7] Siddiqi M.Z., Shafi S.M., Choi K.D., Im W.-T., Aslam Z. (2016). *Sphingobacterium jejuense* sp. nov., with ginsenoside-converting activity, isolated from compost. Int. J. Syst. Evol. Microbiol..

[8] An D.S., Cui C.H., Siddiqi M.Z., Yu H.S., Jin F.X., Kim S.G., Im W.T. (2017). Gram-Scale Production of Ginsenoside F1 Using a Recombinant Bacterial β-Glucosidase. J. Microbiol. Biotechnol..

[9] Christensen L.P. (2009). Ginsenosides chemistry, biosynthesis, analysis, and potential health effects. Adv. Food Nutr. Res..

[10] Jia L., Zhao Y., Liang X.-J. (2009). Current evaluation of the millennium phytomedicine- ginseng (II): Collected chemical entities, modern pharmacology, and clinical applications emanated from traditional Chinese medicine. Curr. Med. Chem..

[11] Mochizuki M., Yoo Y., Matsuzawa K., Sato K., Saiki I., Tonooka S., Samukawa K., Azuma I. (1995). Inhibitory Effect of Tumor Metastasis in Mice by Saponins, Ginsenoside-Rb2, 20(R)- and 20(S)-Ginsenoside-Rg3, of Red ginseng. Boil. Pharm. Bull..

[12] Keum Y.-S., Han S.S., Chun K.-S., Park K.-K., Park J.-H., Lee S.K., Surh Y.-J. (2003). Inhibitory effects of the ginsenoside Rg3 on phorbol ester-induced cyclooxygenase-2 expression, NF-kappaB activation and tumor promotion. Mutat. Res. Mol. Mech. Mutagen..

[13] Han B., Park M., Han Y., Woo L., Sankawa U., Yahara S., Tanaka O. (1982). Degradation of Ginseng Saponins under Mild Acidic Conditions. Planta Medica.

[14] Iishi H., Tatsuta M., Baba M., Uehara H., Nakaizumi A., Shinkai K., Akedo H., Funai H., Ishiguro S., Kitagawa I. (1997). Inhibition by ginsenoside Rg3 of bombesin-enhanced peritoneal metastasis of intestinal adenocarcinomas induced by azoxymethane in Wistar rats. Clin. Exp. Metastasis.

[15] Kim D.G., Jung K.H., Lee D.G., Yoon J.H., Choi K.S., Kwon H.M., Morgan M.J., Howang S.S., Kim Y.S. (2014). 20(S)-Ginsenoside Rg3 is a novel inhibitor of autophagy and sensitizes hepatocellular carcinoma to doxorubicin. Oncotarget.

[16] Kim H.-S., Lee E.-H., Ko S.-R., Choi K.-J., Park J.-H., Im N.-S. (2004). Effects of ginsenosides Rg3 and Rh2 on the proliferation of prostate cancer cells. Arch. Pharmacal Res..

[17] Xu T.-M., Xin Y., Cui M.-H., Jiang X., Gu L.-P. (2007). Inhibitory effect of ginsenoside Rg3 combined with cyclophosphamide on growth and angiogenesis of ovarian cancer. Chin. Med. J..

[18] Tian J., Zhang S., Li G., Liu Z., Xu B. (2009). 20(S)-ginsenoside Rg3, a neuroprotective agent, inhibits mitochondrial permeability transition pores in rat brain. Phytotherapy Res..

[19] In J.G., Kim E.J., Lee B.S., Park M.H., Yang D.C. (2006). Saponin analysis and red ginseng production using the simplified method of Korean ginseng (Panax ginseng C.A. Meyer). Korean J. Plant Res..

[20] Jung H.M., Liu Q.M., Kim J.W., Lee S.T., Kim S.C., Im W.T. (2013). *Lactobacillus ginsenosidimutans* sp. nov., isolated from kimchi with the ability to transform ginsenosides. Antonie van Leeuwenhoek.

[21] Sawatari Y., Hirano T., Yokota A. (2006). Development of food grade media for the preparation of Lactobacillus plantarum starter culture. J. Gen. Appl. Microbiol..

[22] Siddiqi M.Z., Muhammad Shafi S., Im W.T. (2017). Complete genome sequencing of *Arachidicoccus ginsenosidimutans* sp. nov., and its application for production of minor ginsenosides by finding a novel ginsenoside-transforming b-glucosidase. RSC Adv..

[23] Thompson J.D., Gibson T.J., Plewniak F., Jeanmougin F., Higgins D.G. (1997). The CLUSTAL_X windows interface: Flexible strategies for multiple sequence alignment aided by quality analysis tools. Nucleic Acids Res..

[24] Hall T.A. (1999). Bioedit: A user-friendly biological sequence alignment editor and analysis program for Windows 95/98/NT. Nucl. Acids Symp. Ser..

[25] Saitou N., Nei M. (1987). The neighbor-joining method: A new method for reconstructing phylogenetic trees. Mol. Boil. Evol..

[26] Tamura K., Stecher G., Peterson D., Filipski A., Kumar S. (2013). MEGA6: Molecular Evolutionary Genetics Analysis Version 6.0. Mol. Biol. Evol..

[27] Felsenstein J. (1985). Confidence limits on phylogenies: An approach using the bootstrap. Evolution.

[28] Ruan C.-C., Zhang H., Zhang L.-X., Liu Z., Sun G.-Z., Lei J., Qin Y.-X., Zheng Y.-N., Li X., Pan H.-Y. (2009). Biotransformation of Ginsenoside Rf to Rh1 by Recombinant β-Glucosidase. Molecules.

[29] Choi S.-H., Shin T.-J., Hwang S.-H., Lee B.-H., Kang J.-Y., Kim H.-J., Oh J.-W., Bae C.-S., Lee S.-H., Nah S.-Y. (2011). Differential Effects of Ginsenoside Metabolites on HERG K+ Channel Currents. J. Ginseng Res..

[30] Siddiqi M.Z., Cui C.H., Park S.K., Han N.S., Kim S.C., Im W.T. (2017). Comparative analysis of the expression level of recombinant ginsenoside-transforming β-glucosidase in GRAS hosts and mass production of the ginsenoside Rh2-Mix. PLoS ONE.

[31] Li L., Lee S.J., Yuan Q.P., Im W.T., Kim S.C., Han N.N. (2018). Production of bioactive ginsenoside Rg3(S) and compound K using recombinant Lactococcus lactis. J. Ginseng Res..

[32] Cheng L.-Q., Na J.R., Bang M.H., Kim M.K., Yang D.-C. (2008). Conversion of major ginsenoside Rb1 to 20(S)-ginsenoside Rg3 by Microbacterium sp. GS514. Phytochemistry.

[33] Quan L.H., Min J.W., Yang D.U., Kim Y.J., Yang D.C. (2012). Enzymatic biotransformation of ginsenoside Rb1 to 20(S)-Rg3 by recombinant bglucosidase from Microbacterium esteraromaticum. Appl. Micro Biotech..

[34] Opassiri R., Pomthong B., Onkoksoong T., Akiyama T., Esen A., Cairns J.R.K. (2006). Analysis of rice glycosyl hydrolase family 1 and expression of Os4bglu12 β-glucosidase. BMC Plant Boil..

[35] An D.-S., Cui C.-H., Lee H.-G., Wang L., Kim S.C., Lee S.-T., Jin F., Yu H., Chin Y.-W., Lee H.-K. (2010). Identification and Characterization of a Novel Terrabacter ginsenosidimutans sp. nov. β-Glucosidase That Transforms Ginsenoside Rb1 into the Rare Gypenosides XVII and LXXV. Appl. Environ. Microbiol..

