# Supplementary material for: Exploration and Characterization of Novel Glycoside Hydrolases from the Whole Genome of Lactobacillus ginsenosidimutans and Enriched Production of Minor Ginsenoside Rg3(S) by a Recombinant Enzymatic Process
Source: Biomolecules. 2020 Feb 12;10(2):288. doi: 10.3390/biom10020288 (PMC7072194; doi:10.3390/biom10020288)
Supplement: Supplementary file 1 [file biomolecules-10-00288-s001.pdf]

biomolecules

Exploration and Characterization of Novel Glycoside Hydrolases from the Whole Genome of *Lactobacillus ginsenosidimutans* and Enriched Production of Minor Ginsenoside Rg3(*S*) by a Recombinant Enzymatic Process

Muhammad Zubair Siddiqi [1,2], Sathiyaraj Srinivasan [3], Hye Yoon Park [4] and Wan-Taek Im [1,2,*]

[1] Department of Biotechnology, Hankyong National University, 327 Jungang-ro Anseong-si, Gyeonggi-do 17579, Korea; mzsiddiqi1988@gmail.com

[2] AceEMzyme Co., Ltd., Academic Industry Cooperation, 327 Jungang-ro Anseong-si, Gyeonggi-do Anseong-si, Gyeonggi-do 17579, Korea

[3] Department of Bio & Environmental Technology, Division of Environmental & Life Science, College of Natural Science, Seoul Women's University, 623 Hwarangno, Nowon-gu, Seoul 139-774, Republic of Korea; sathiya.micro@gmail.com

[4] National Institute of Biological Resources, Incheon 22689, Korea; rejoice077@korea.kr

***Correspondence: wandra@hknu.ac.kr; Tel.: +82-31-6705335; Fax: +82-31-6705339**

Figure S1.

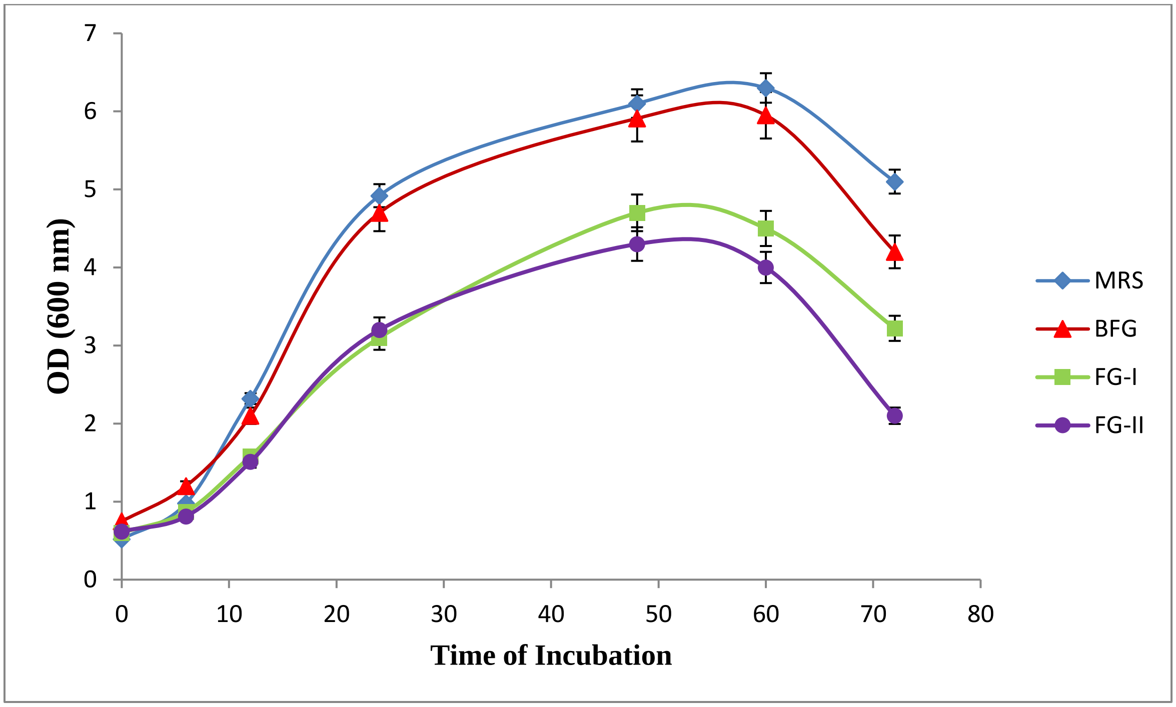

Figure S1. Growth profile of L. ginsenosidimutans in MRS and three food grade media [Based Food Grade (BFG), Food Grade-I (FG-I) and Food Grade-II (FG-II)]. Growth curves were performed in triplicate and the average of those measurements is displayed in the graph above.

Fig. S2

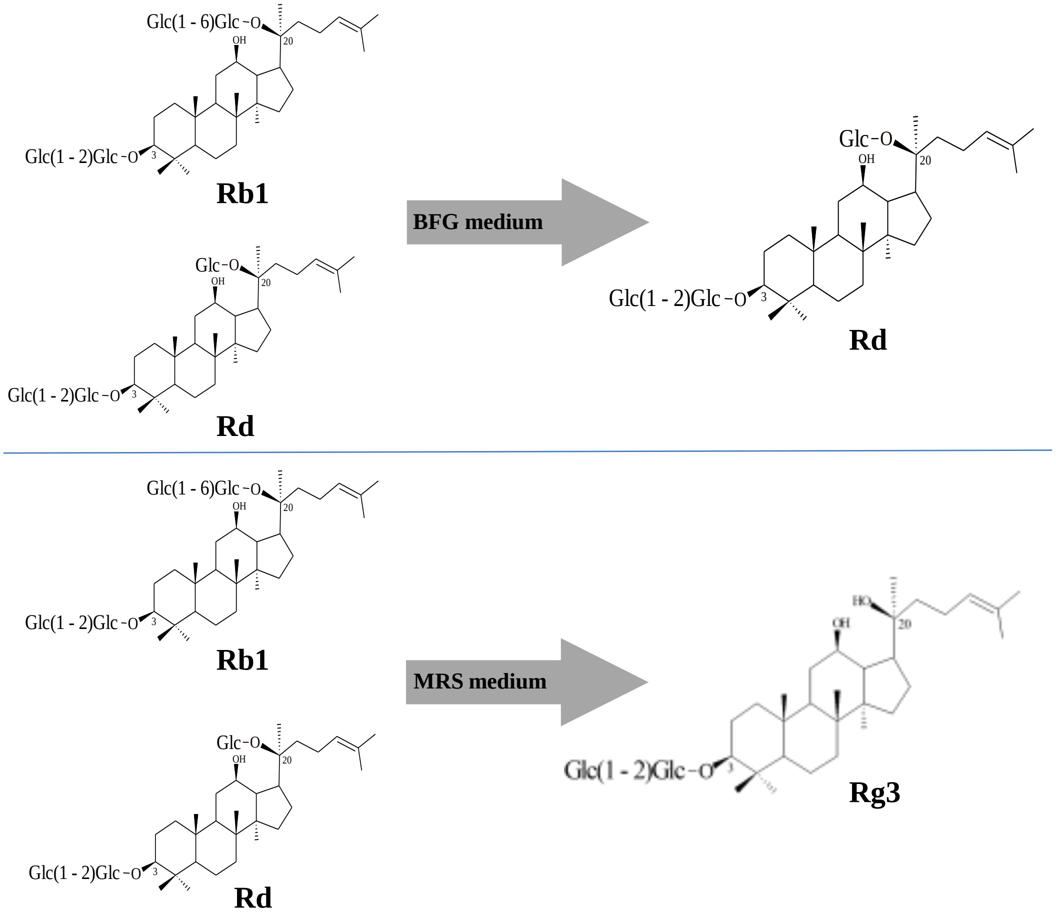

Figure S2. Show the conversion of PPD-mix type major ginsenoside (Rb1and Rd) to Rg3 by L. ginsenosidimutans EMML 3041T.

Fig. S3

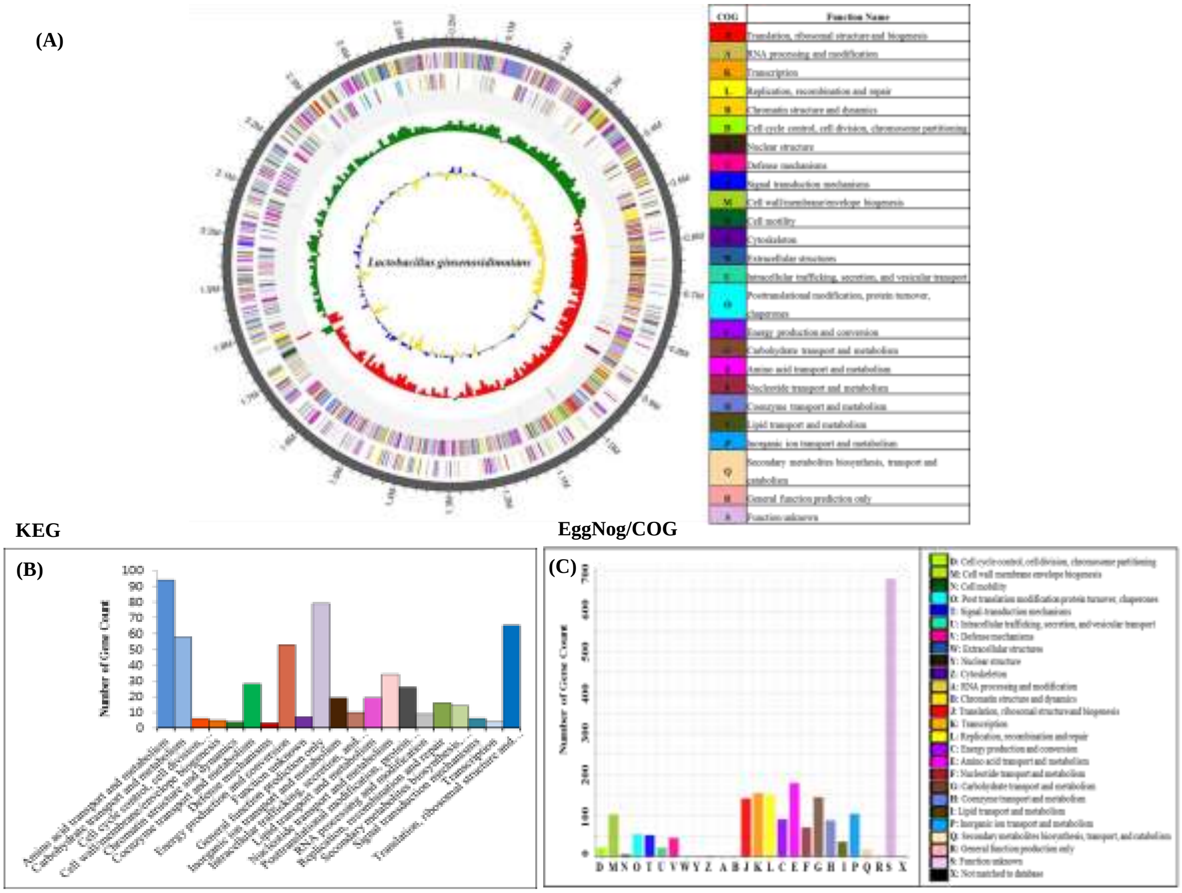

Figure S3. Graphical map of the chromosome of Lactobacillus ginsenosidimutans EMML 3041T. From outside to the center: Genes on the forward strand (colored by COG categories), genes on the reverse strand (colored by COG categories), RNA genes (tRNAs, green; rRNAs, red; other RNAs, black), and GC content and GC skew. And the orthologous proteins of Lactobacillus ginsenosidimutans EMML 3041T and distribution of proteins in different KEG/COG categories are plotted. Y-axis represents the number of genes/proteins and X-axis represents the KEG/COG categories.

Figure S4

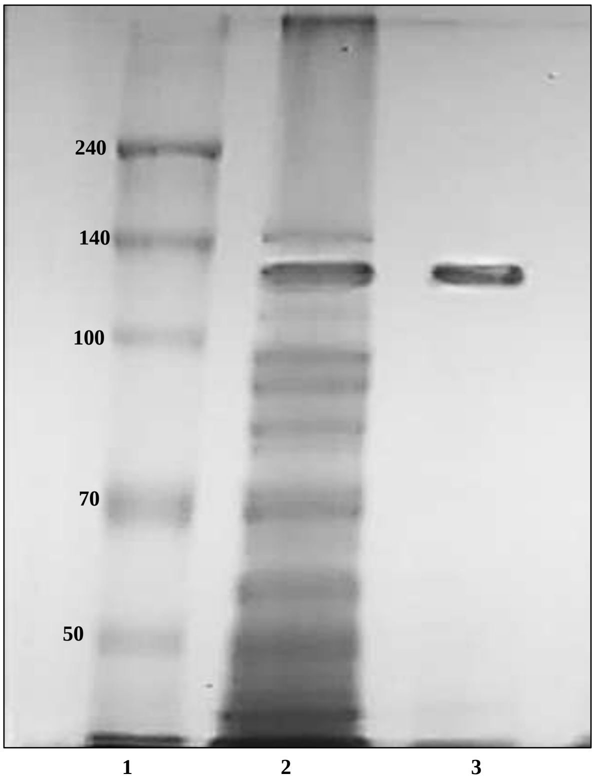

Figure S4. SDS page analyses of purified BglL.gin-952. Lane 1, Protein marker; Len 2, Induced BglL.gin-952; Lane 3, purified BglL.gin-952.

Figure S5

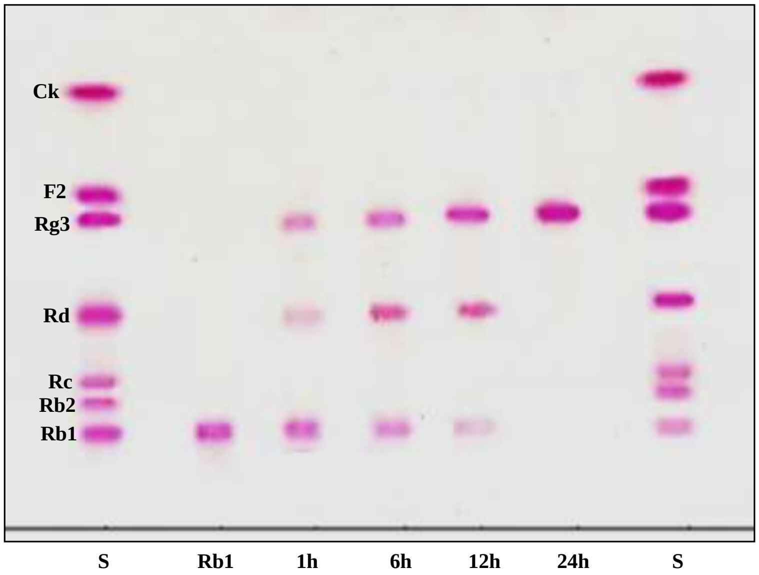

Figure S5. TLC analyses of time course of bioconversion of ginsenosides Rb1 by BglL.gin-952. S, ginsenosides standards.

Table S1. Anticancer activities of ginsenoside Rg3.

Types of cancers	Ginsenoside	Study *In vitro*/ *in vivo*	Cell type	Effects	Mechanisms	References
Breast	Rg3	*In vitro* (30 μM)	MDA-MB-231	Induction of apoptosis	Inhibition of mutant p53 apoptosis and NF-κB signaling via possibly inactivation of ERK and Akt to activate mitochondrial death pathway	[1]
Breast	20(*S*)Rg3	*In vitro* (100-300 μM)	MCF-7 MDA-MB-231	Inhibition of proliferation	Arrested the cells in the G1-phase	[2]
Colon	20(*S*)Rg3	*In vitro* (≥100 μM)	HT-29	Inhibition of proliferation, induction of apoptosis	Reduced PCNA, STRAP and other protein related induction of to mitosis and DNA repair, apoptosis downregulated Rho-GDI while upregulated TM1, GSTP1 and Annexin	[3]
Colon	Rg3	*In vitro* ≥100 μM/ *in vivo* (20mg/kg)	HCT116 SW480	Inhibition of proliferation, Inhibition of tumor growth in xenograft model	Blocked the nuclear translocation of β-catenin and then inhibit β-catenin/Tcf signal pathway	[4]
Colon	Rg3	*In vitro* (200 μM)	SW480	Inhibition of metastasis	Inhibited NF-κB signaling pathway and the NF-κB-regulated gene expression such as c-Myc, COX-2 and MMP-9	[5]
Esophageal carcinoma Renal	Rg3	In vitro (25-200 μM)	Eca-109 786-0	Inhibition of proliferation and angiogenesis	Suppressed VEGF expression by blocking multiple signaling pathways including HIF-1α, COX-2, NF-κB, STAT3 and MAPKs	[6]
Gallbladder	Rg3	*In vitro* (10-100 μM)/*in vivo* (20mg/kg)	GBC-SD QBC939	Inhibition of proliferation and tumor growth in	Activation of ER stress to regulate apoptosis related proteins such as caspase-12, CHOP	[7]

				xenograft model		
Glioblastoma multiforme	Rg3	*In vitro* (≥10 μM)	U87MG	Induction of apoptosis	Suppression of MEK/MAPK signaling pathway, activation of ROS by antioxidant enzyme system	[8]
Gastric	20(*S*)-Rg3	*In vitro* (25-100 μM)	AGS	Inhibition of proliferation, induction of apoptosis	Upregulation of caspase-3, caspase-8, caspase-9 and Bax while downregulation of Bcl-2	[9]
Gastric	Rg3	*In vitro* (50 μg/ml)	SGC-7901	Induction of apoptosis	Inhibition of the expression of FUT4 via SP1 and HSF1 transcriptional regulation and eventually, activation of caspase-3, caspase-8 and caspase-9	[10]
Ovarian	20(S)-Rg3	*In vitro*/ *in vivo*	SKOV-3 (160 μg/ml)/ 3AO (80 μg/ml)	Induction of apoptosis, inhibition of proliferation, inhibition of the growth of tumor xenografts	Suppression of Warburg effect though the STAT3/HK2 pathway	[11]
Pancreatic	Rg3	In vitro (25-200 μM)/ in vivo (5, 10, 20 mg/kg)	SW-1990	Inhibition of VM and the growth of tumor xenografts	Inhibition of the expression of particular genes including VE-cadherin, EphA2, MMP-2 and MMP-9	[12]
Prostate	20(S)-Rg3	In vitro (1-10 μM)	PC-3M	Inhibition of migration	Transcriptionally inhibited the expression of AQP1 via the p38 MAPK pathway	[13]

Table S2. Show the composition of MRS (Difco), Basel Food Grade medium (BFG), Food Grade medium I and Food Grade medium II. All ingredients were used as g/L.

**Composition (g/L)**	**MRS**	**Basel FG**	**FG-I**	**FG-II**
Glucose	20.0	-	-	-
Table sugar	-	20.0	20.0	20.0
Bacto Proteose Peptone No. 3	10.0	-	-	-
Bacto Beef Extract	10.0	-	-	-
Bacto Yeast Extract	5.0	-	-	-
Yeast extract/Peptone	-	10/20	10/20	10/20
Tween 80 (mL)	1.0	-	-	-
Sorbitan oleate Span® 80 (mL)	-	0.75	0.75	0.75
K_2HPO_4	2.0	2.0	-	-
Sodium acetate	5.0	5.0	5.0	-
Triammonium citrate	2.0	-	-	-
Trisodium citrate	-	2.1	2.1	-
$MgSO_4 \cdot 7H_2O$	0.1	0.1	-	-
$MnSO_4 \cdot 4–5H_2O$	0.05	0.05	0.05	0.05

Table S3. Identification of glycoside hydrolases from the whole genome sequence of L. ginsenosidimutans EMML 3041T.

S.No	Name	Product	Amino acids	COG ID	Function
1	GCA_001050475.1_02325	D-Ribose pyranase	396	COG1869	Catalyzes the interconversion of β-pyran andβ-furan forms of D-ribose (By similarity)
2	GCA_001050475.1_01525	UDP-Galactopyranose mutase	1122	COG0562	Hydrolyse the beta-pyran and β-furan forms
3	GCA_001050475.1_01123	Endo-1,4-β-xylanase	825	COG0657	Carbohydrate metabolism; Glycosidase; Hydrolase; Polysaccharide degradation; Xylan degradation
4	GCA_001050475.1_02134	Oligo-1,6-Glucosidase	1671	COG0366	Glycosidase; Hydrolase, Belongs to the glycosyl hydrolase 13 family
5	GCA_001050475.1_02147	β-D-Ribofuranosyl	804	COG1235	Hydrolase; Metal-binding. Belongs to the metallo-β-lactamase superfamily

Table S4. Show the hydrolytic activities of seven glycoside hydrolase clones of L. ginsenosidimutans EMML 3041T for various sugar substrates.

1, *α*-glucosidase-556; 2, β-glucosidase-484; 3, β-glucosidase-902; 4, β-glucosidase-952; 5, *α*-galactosidase-556; 6, *α*-galactosidase-319; 7, β-galactosidase-629. ++, strong positive (98.0 ± 1.2 %); +, positive (87.0 ± 3.1 %); w, weak positive (63.0 ± 2.2 %); –, negative.

**Substrates**	**Clones of *L. ginsenosidimutans* EMML 3041^T**						
	**1**	**2**	**3**	**4**	**5**	**6**	**7**
*p*NP-*β*-D-Glucopyranoside	+	+	w	++	w	+	+
*p*NP-*β*-D-Galactopyranoside	–	–	–	–	–	w	w
*p*NP-*β*-D-Fucopyranoside	–	–	–	–	–	–	–
*p*NP-*β*-D-Glucosaminide	–	–	–	–	–	–	–
*p*NP-*β*-L-Arabinofuranoside	–	–	–	–	–	–	–
*p*NP-*β*-Mannopyranoside	–	–	–	–	–	–	–
*p*NP-*β*-D-Xylopyranoside	–	–	–	–	–	–	–
*p*NP-*α*-D-Glucopyranoside	–	–	–	–	–	–	–
*p*NP-*α*-L-Arabinofuranoside	–	–	–	–	–	–	–
*p*NP-*α*-D-Fucopyranoside	–	–	–	–	–	–	–
*p*NP-*α*-L-Rhamnnopyranoside	–	–	–	–	–	–	–
*p*NP-*α*-D-Mannopyranoside	–	–	–	–	–	–	–
*o*NP-*β*-D-Glucopyranoside	w	+	+	–	+	–	w
*o*NP-*β*-D-Fucopyranoside	–	–	–	–	–	–	–
*o*NP-*β*-D-Galactopyranoside	–	–	–	–	–	–	w
**Bioconversion of ginsenosides**	–	–	–	+	–	–	–

Table S5. Show the different sugar substrate used for the BglL.gin-952.

S.No	Various kind of sugar Substrates
1	*p*NP-β-D-glucopyranoside
2	*p*NP-β-D-galactopyranoside
3	*p*NP-β-D-fucopyranoside
4	*p*NP-β-N-glucosaminide
5	*p*NP-β-L-arabinopyranoside
6	*p*NP-β-D-mannopyranoside
7	*p*NP-β-D-xylopyranoside
8	*p*NP-α-D-glucopyranoside
9	*p*NP-α-L-arabinofuranoside
10	*p*NP-α-L-arabinopyranoside
11	*p*NP-α-L-mannopyranoside
12	*p*NP-α-D-mannopyranoside
13	*o*NP-β-D-glucopyranoside
14	*o*NP-β-D-fucopyranoside
15	*o*NP-α-D-galactopyranoside

Table S6. Total gene count and their function.

Function	Gene count	%age
Amino acid transport and metabolism	94	16.82%
Carbohydrate transport and metabolism	58	10.38%
Cell cycle control, cell division, chromosome partitioning	6	1.07%
Cell wall/membrane/envelope biogenesis	5	0.89%
Chromatin structure and dynamics	4	0.72%
Coenzyme transport and metabolism	28	5.01%
Defense mechanisms	3	0.54%
Energy production and conversion	53	9.48%
Function unknown	7	1.25%
General function prediction only	79	14.13%
Inorganic ion transport and metabolism	19	3.40%
Intracellular trafficking, secretion, and vesicular transport	10	1.79%
Lipid transport and metabolism	19	3.40%
Nucleotide transport and metabolism	34	6.08%
Posttranslational modification, protein turnover, chaperones	26	4.65%
RNA processing and modification	9	1.61%
Replication, recombination and repair	16	2.86%
Secondary metabolites biosynthesis, transport and catabolism	14	2.50%
Signal transduction mechanisms	6	1.07%
Transcription	4	0.72%
Translation, ribosomal structure and biogenesis	65	11.63%
Not in KOG	2101	80.04%

Table S7. The total housekeeping gene identified in the whole genome sequenced L. ginsenosidimutans EMML 3041T.

S.No	Gene ID	Housekeeping gene	Product name	Description
1	TIGR00133	gatB	Protein synthesis	aspartyl/glutamyl-tRNA(Asn/Gln) amidotransferase, B subunit
2	TIGR03953	rplD_bact	Protein synthesis	50S ribosomal protein uL4
3	TIGR00135	gatC	Protein synthesis	aspartyl/glutamyl-tRNA(Asn/Gln) amidotransferase, C subunit
4	Methyltransf_5	PF01795.15	Unclassified	MraW methylase family
5	TIGR02027	rpoA	Transcription	DNA-directed RNA polymerase, alpha subunit
6	TIGR00334	5S_RNA_mat_M5	Transcription	ribonuclease M5
7	TIGR01044	rplV_bact	Protein synthesis	ribosomal protein uL22
8	TIGR00653	GlnA	Amino acid biosynthesis	glutamine synthetase, type I
9	TIGR01146	ATPsyn_F1gamma	Energy metabolism	ATP synthase F1, gamma subunit
10	TIGR00967	3a0501s007	Protein fate	preprotein translocase, SecY subunit

11	TIGR00755	ksgA	Protein synthesis	ribosomal RNA small subunit methyltransferase A
12	TIGR00450	mnmE_trmE_thdF	Protein synthesis	tRNA modification GTPase TrmE
13	TIGR00456	argS	Protein synthesis	arginine--tRNA ligase
14	TIGR00615	recR	DNA metabolism	recombination protein RecR
15	TIGR03598	GTPase_YsxC	Protein synthesis	ribosome biogenesis GTP-binding protein YsxC
16	TIGR00082	rbfA	Transcription	ribosome-binding factor A
17	TIGR02348	GroEL	Protein fate	chaperonin GroL
18	TIGR00246	tRNA_RlmH_YbeA	Protein synthesis	rRNA large subunit m3Psi methyltransferase RlmH
19	TIGR01133	murG	Cell envelope	undecaprenyldiphospho-muramoylpentapeptide beta-N-acetylglucosaminyltransferase
20	TIGR01135	glmS	Central intermediary metabolism	glutamine-fructose-6-phosphate transaminase (isomerizing)
21	TIGR01031	rpmF_bact	Protein synthesis	ribosomal protein bL32
22	TIGR00019	prfA	Protein synthesis	peptide chain release factor 1

23	TIGR00012	L29	Protein synthesis	ribosomal protein uL29
24	TIGR00150	T6A_YjeE	Protein synthesis	tRNA threonylcarbamoyl adenosine modification protein YjeE
25	TIGR00152	TIGR00152	Biosynthesis of cofactors, prosthetic groups, and carriers	dephospho-CoA kinase
26	TIGR00981	rpsL_bact	Protein synthesis	ribosomal protein uS12
27	TIGR00091	TIGR00091	Protein synthesis	tRNA (guanine-N(7)-)-methyltransferase
28	TIGR00095	TIGR00095	Protein synthesis	16S rRNA (guanine(966)-N(2))-methyltransferase RsmD
29	TIGR00256	TIGR00256	Protein synthesis	D-tyrosyl-tRNA(Tyr) deacylase
30	TIGR01306	GMP_reduct_2	Purines, pyrimidines, nucleosides, and nucleotides	guanosine monophosphate reductase
31	TIGR01144	ATP_synt_b	Energy metabolism	ATP synthase F0, B subunit

32	TIGR01029	rpsG_bact	Protein synthesis	ribosomal protein uS7
33	TIGR00382	clpX	Protein fate	ATP-dependent Clp protease, ATP-binding subunit ClpX
34	TIGR00065	ftsZ	Cellular processes	cell division protein FtsZ
35	TIGR01394	TypA_BipA	Regulatory functions	GTP-binding protein TypA/BipA
36	TIGR01743	purR_Bsub	Regulatory functions	pur operon repressor PurR
37	TIGR00435	cysS	Protein synthesis	cysteine--tRNA ligase
38	TIGR00635	ruvB	DNA metabolism	Holliday junction DNA helicase RuvB
39	TIGR00431	TruB	Protein synthesis	tRNA pseudouridine(55) synthase
40	TIGR01091	upp	Purines, pyrimidines, nucleosides, and nucleotides	uracil phosphoribosyltransferase
41	TIGR01090	apt	Purines, pyrimidines, nucleosides, and nucleotides	adenine phosphoribosyltransferase

42	TIGR01017	rpsD_bact	Protein synthesis	ribosomal protein uS4
43	TIGR01011	rpsB_bact	Protein synthesis	ribosomal protein uS2
44	TIGR00079	pept_deformyl	Protein fate	peptide deformylase
45	TIGR00071	hisT_truA	Protein synthesis	tRNA pseudouridine(38-40) synthase
46	TIGR02397	dnaX_nterm	DNA metabolism	DNA polymerase III, subunit gamma and tau
47	TIGR00647	DNA_bind_WhiA	Cellular processes	DNA-binding protein WhiA
48	TIGR00484	EF-G	Protein synthesis	translation elongation factor G
49	TIGR00179	murB	Cell envelope	UDP-N-acetylenolpyruvoylglucosamine reductase
50	TIGR01087	murD	Cell envelope	UDP-N-acetylmuramoylalanine--D-glutamate ligase
51	TIGR01169	rplA_bact	Protein synthesis	ribosomal protein uL1
52	TIGR00487	IF-2	Protein synthesis	translation initiation factor IF-2
53	TIGR01164	rplP_bact	Protein synthesis	ribosomal protein uL16
54	TIGR01009	rpsC_bact	Protein synthesis	ribosomal protein uS3

55	TIGR00041	DTMP_kinase	Purines, pyrimidines, nucleosides, and nucleotides	dTMP kinase
56	TIGR01128	holA	DNA metabolism	DNA polymerase III, delta subunit
57	TIGR01455	glmM	Central intermediary metabolism	phosphoglucosamine mutase
58	TIGR00166	S6	Protein synthesis	ribosomal protein bS6
59	TIGR02075	pyrH_bact	Purines, pyrimidines, nucleosides, and nucleotides	UMP kinase
60	TIGR00419	tim	Energy metabolism	triose-phosphate isomerase
61	TIGR00496	frr	Protein synthesis	ribosome recycling factor
62	TIGR03654	L6_bact	Protein synthesis	ribosomal protein uL6
63	TIGR01171	rplB_bact	Protein synthesis	ribosomal protein uL2
64	TIGR00492	alr	Cell envelope	alanine racemase
65	TIGR00810	secG	Protein fate	preprotein translocase, SecG subunit

66	TIGR01071	rplO_bact	Protein synthesis	ribosomal protein uL15
67	TIGR00054	TIGR00054	Protein fate	RIP metalloprotease RseP
68	TIGR00055	uppS	Cell envelope	di-trans,poly-cis-decaprenylcistransferase
69	TIGR01704	MTA/SAH-Nsdase	Purines, pyrimidines, nucleosides, and nucleotides	MTA/SAH nucleosidase
70	TIGR00115	tig	Protein fate	trigger factor
71	TIGR00059	L17	Protein synthesis	ribosomal protein bL17
72	TIGR00593	pola	DNA metabolism	DNA polymerase I
73	TIGR00464	gltX_bact	Protein synthesis	glutamate--tRNA ligase
74	TIGR01066	rplM_bact	Protein synthesis	ribosomal protein uL13
75	Ribosomal_S8	PF00410.15	Unclassified	Ribosomal protein S8
76	TIGR00105	L31	Protein synthesis	ribosomal protein bL31
77	TIGR02729	Obg_CgtA	Protein synthesis	Obg family GTPase CgtA
78	TIGR03534	RF_mod_PrmC	Protein fate	protein-(glutamine-N5) methyltransferase, release factor-specific

79	TIGR00029	S20	Protein synthesis	ribosomal protein bS20
80	TIGR03635	uS17_bact	Protein synthesis	ribosomal protein uS17
81	TIGR01059	gyrB	DNA metabolism	DNA gyrase, B subunit
82	TIGR03631	uS13_bact	Protein synthesis	ribosomal protein uS13
83	TIGR03632	uS11_bact	Protein synthesis	ribosomal protein uS11
84	TIGR01050	rpsS_bact	Protein synthesis	ribosomal protein uS19
85	TIGR00188	rnpA	Transcription	ribonuclease P protein component
86	TIGR00873	gnd	Energy metabolism	6-phosphogluconate dehydrogenase (decarboxylating)
87	TIGR00871	zwf	Energy metabolism	glucose-6-phosphate dehydrogenase

Table S8. Show the production of Rg3(S) using the Lac. ginsenosidimutans EMML 3041T and recombinant BglLgin-952.

Starting substrate	Weight of substrate (g/L)	Strain/Recombinant enzyme	Media	Molar ration of Rg3	Purity (%)	Reaction time	Uses
PPD-mix (Rb1, and Rd)	5	*Lac. ginsenosidimutans*	MRS	0.00127	53.1	72hrs	Food supplement
		BglL.gin-952	LB ampicillin	0.00382	68.5	24hrs	Pharmaceutical
Rb1 (purity ≥ 80.0)	10	BglL.gin-952	LB ampicillin	0.0076	74.3	24hrs	Pharmaceutical

Table S1, References:

1. Kim, B.-M.; Kim, D.-H.; Park, J.-H.; Surh, Y.-J.; Na, H.-K. Ginsenoside Rg3 Inhibits Constitutive Activation of NF-κB Signaling in Human Breast Cancer (MDA-MB-231) Cells: ERK and Akt as Potential Upstream Targets. *J. Cancer Prev.* **2014**, *19*, 23–30.
2. Wang, C.-Z.; Aung, H.H.; Zhang, B.; Sun, S.; Li, X.-L.; He, H.; Xie, J.-T.; He, T.-C.; Du, W.; Yuan, C.-S. Chemopreventive effects of heat-processed Panax quinquefolius root on human breast cancer cells. *Anticancer Res.* **2008**, *28*, 2545–2551.
3. Lee, S.Y.; Kim, G.T.; Roh, S.H.; Song, J.-S.; Kim, H.-J.; Hong, S.-S.; Kwon, S.W.; Park, J.H. Proteomic Analysis of the Anti-Cancer Effect of 20S-Ginsenoside Rg3in Human Colon Cancer Cell Lines. *Biosci. Biotechnol. Biochem.* **2009**, *73*, 811–816.
4. He, B.C.; Gao, J.L.; Luo, X.; Luo, J.; Shen, J.; Wang, L.; Zhou, Q.; Wang, Y.T.; Luu, H.H.; Haydon, R.C.; et al. Ginsenoside Rg3 inhibits colorectal tumor growth through the down-regulation of Wnt/β-catenin signaling. *Int. J. Oncol.* **2011**, *38*, 437–445.
5. Junmin, S.; Hongxiang, L.; Zhen, L.; Chao, Y.; Chaojie, W. Ginsenoside Rg3 inhibits colon cancer cell migration by suppressing nuclear factor kappa B activity. *J. Tradit. Chin. Med.* **2015**, *35*, 440–444.
6. Chen, Q.-J.; Zhang, M.-Z.; Wang, L.-X. Gensenoside Rg3 Inhibits Hypoxia-induced VEGF Expression in Human Cancer Cells. *Cell. Physiol. Biochem.* **2010**, *26*, 849–858.
7. Wu, K.; Li, N.; Sun, H.; Xu, T.; Jin, F.; Nie, J. Endoplasmic reticulum stress activation mediates Ginseng Rg3-induced anti-gallbladder cancer cell activity. *Biochem. Biophys. Res. Commun.* **2015**, *466*, 369–375.
8. Choi, Y.J.; Lee, H.J.; Kang, N.W.; Han, I.H.; Choi, B.K.; Cho, W.H. Ginsenoside Rg3 induces apoptosis in the U87MG human glioblastoma cell line through the MEK signaling pathway and reactive oxygen species. *Oncol. Rep.* **2013**, *30*, 1362–1370.
9. Park, E.H.; Kim, Y.J.; Yamabe, N.; Park, S.H.; Kim, H.K.; Jang, H.J.; Kim, J.H.; Cheon, G.J.; Ham, J.; Kang, K.S. Stereospecific anticancer effects of ginsenoside Rg3 epimers isolated from heat-processed American ginseng on human gastric cancer cell. *J. Ginseng Res.* **2014**, *38*, 22–27.
10. Aziz, F.; Wang, X.; Liu, J.; Yan, Q. Ginsenoside Rg3 induces FUT4-mediated apoptosis in H. pylori CagA-treated gastric cancer cells by regulating SP1 and HSF1 expressions. *Toxicol. Vitr.* **2016**, *31*, 158–166.
11. 41. Li, J.; Liu, T.; Zhao, L.; Chen, W.; Hou, H.; Ye, Z.; Li, X. Ginsenoside 20(S) Rg3 inhibits the Warburg effect through STAT3 pathways in ovarian cancer cells. *Int. J. Oncol.* **2015**, *46*, 775–781.
12. Guo, J.-Q.; Zheng, Q.-H.; Chen, H.; Chen, L.; Xu, J.-B.; Chen, M.-Y.; Lu, D.; Wang, Z.-H.; Tong, H.-F.; Lin, S. Ginsenoside Rg3 inhibition of vasculogenic mimicry in pancreatic cancer through downregulation of VE-cadherin/EphA2/MMP9/MMP2 expression. *Int. J. Oncol.* **2014**, *45*, 1065–1072.
13. Pan, X.-Y.; Guo, H.; Han, J.; Hao, F.; An, Y.; Xu, Y.; Xiaokaiti, Y.; Pan, Y.; Li, X.-J. Ginsenoside Rg3 attenuates cell migration via inhibition of aquaporin 1 expression in PC-3M prostate cancer cells. *Eur. J. Pharmacol.* **2012**, *683*, 27–34.
